# Supplementary figures and images for: Membrane binding properties of the cytoskeletal protein bactofilin
Source: eLife. 2025 Sep 19;13:RP100749. doi: 10.7554/eLife.100749 (PMC12448750; doi:10.7554/eLife.100749)

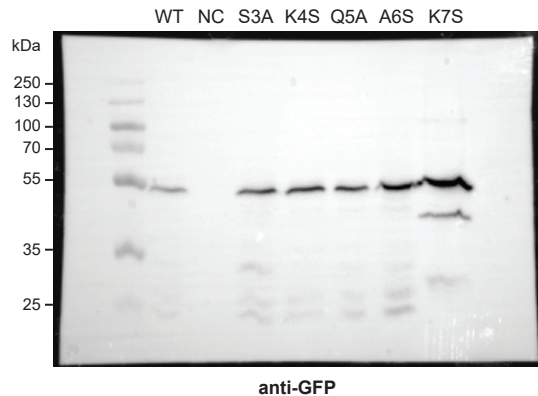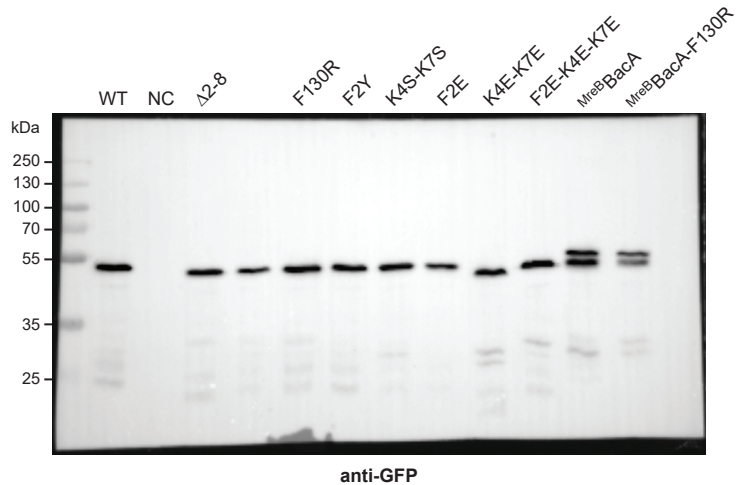

Supplement: Figure 1—figure supplement 3—source data 1. [file elife-100749-fig1-figsupp3-data1.pdf]

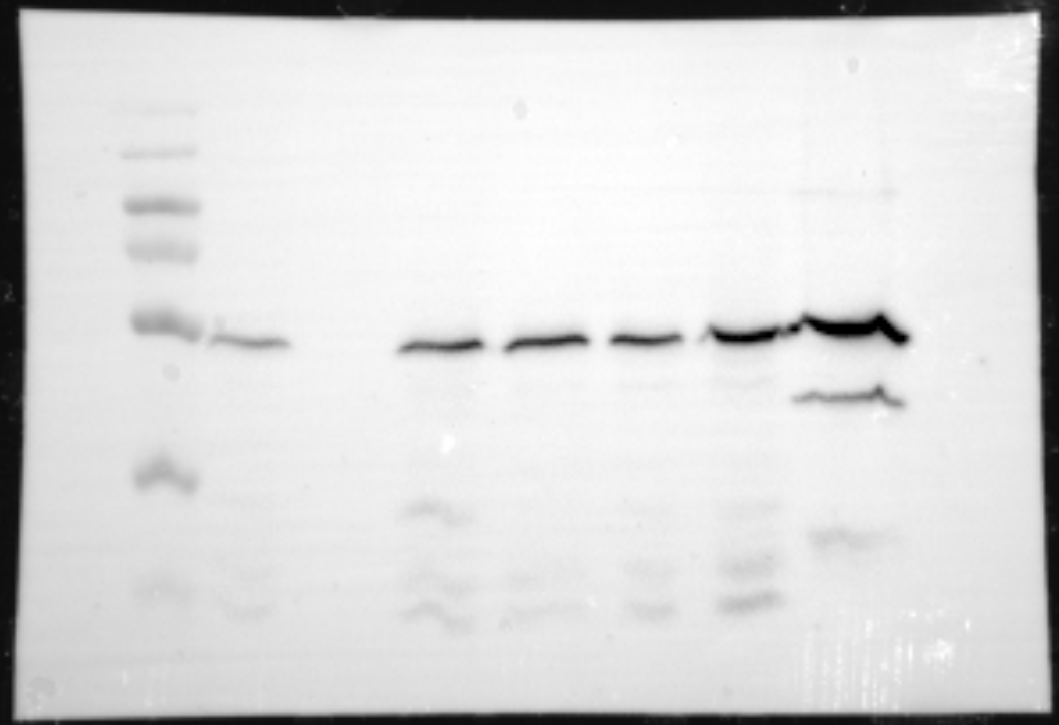

Supplement: Figure 1—figure supplement 3—source data 2. [file elife-100749-fig1-figsupp3-data2.zip › Figure 1-figure supplement 3-source data-left.tif]

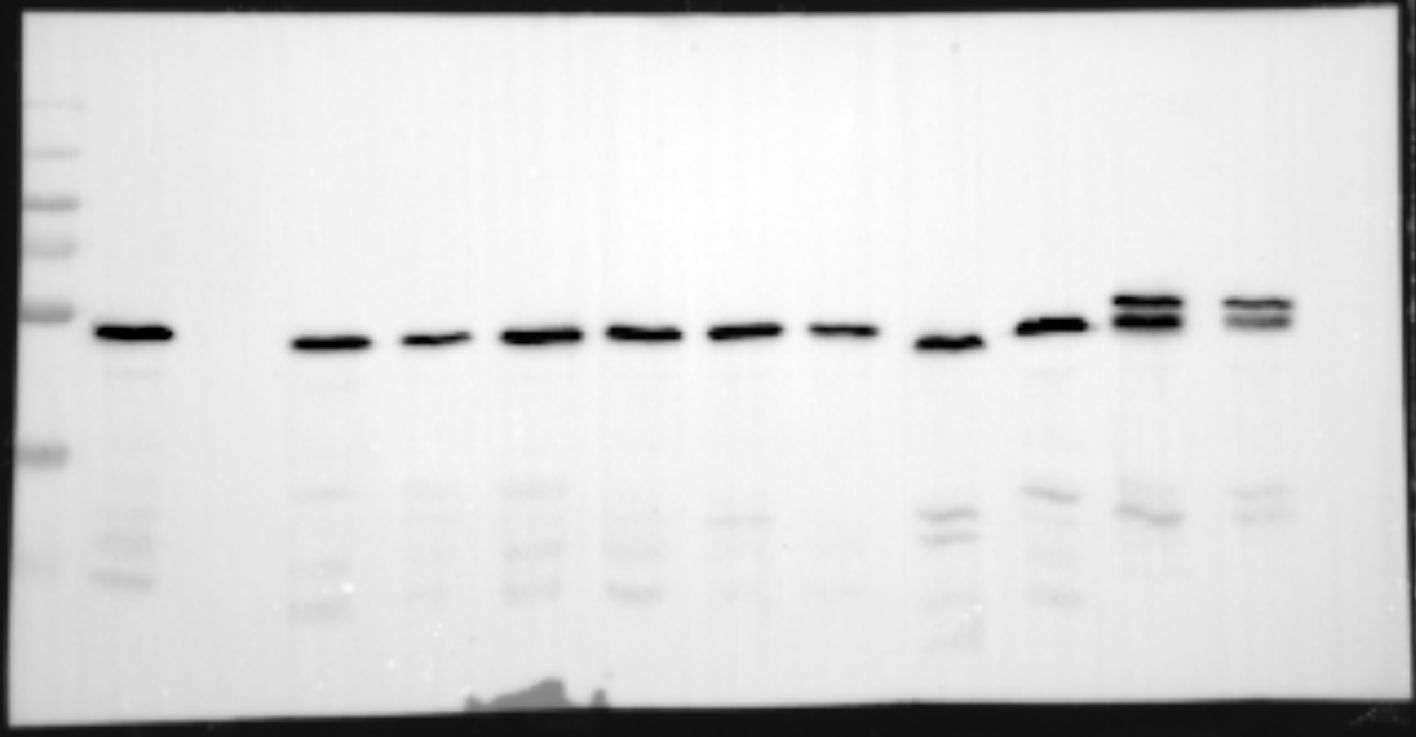

Supplement: Figure 1—figure supplement 3—source data 2. [file elife-100749-fig1-figsupp3-data2.zip › Figure 1-figure supplement 3-source data-right.tif]

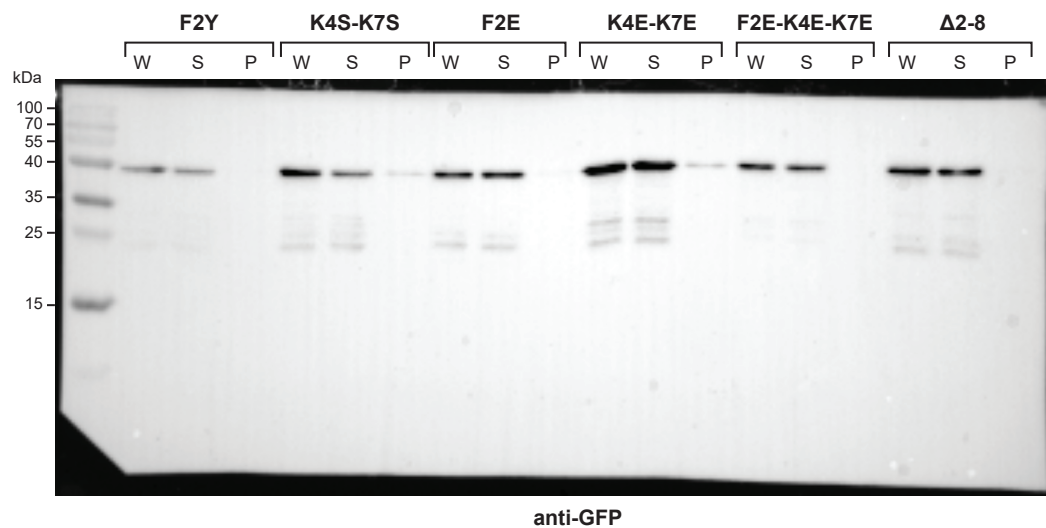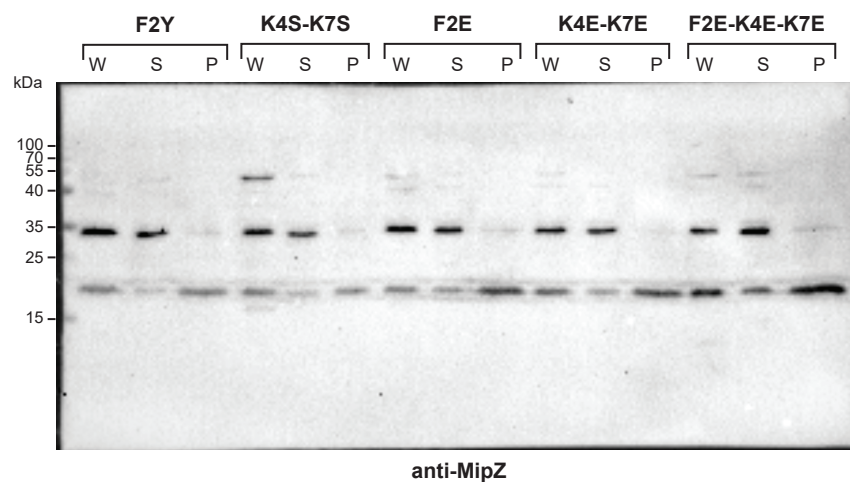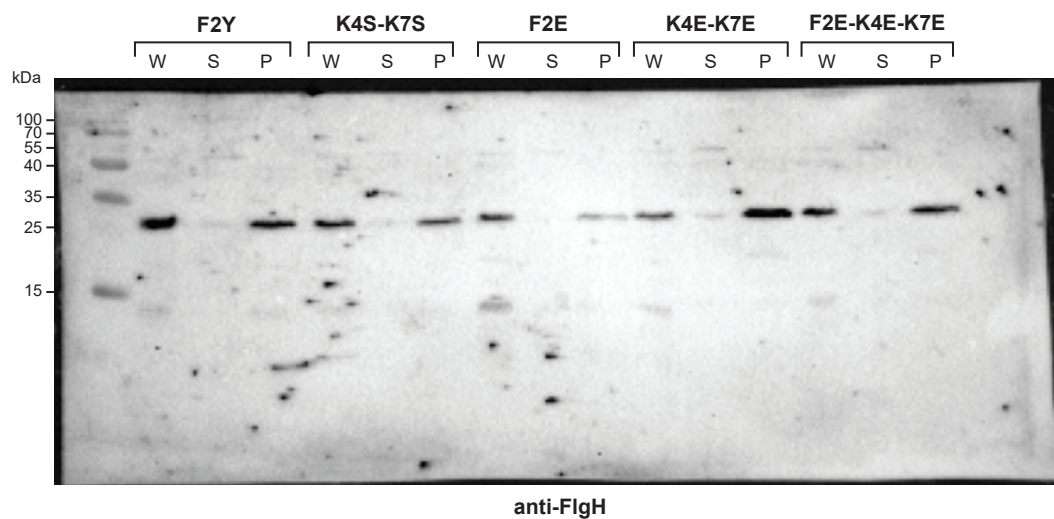

Supplement: Figure 2—source data 1. [file elife-100749-fig2-data1.zip › Figure 2C-source data.pdf]

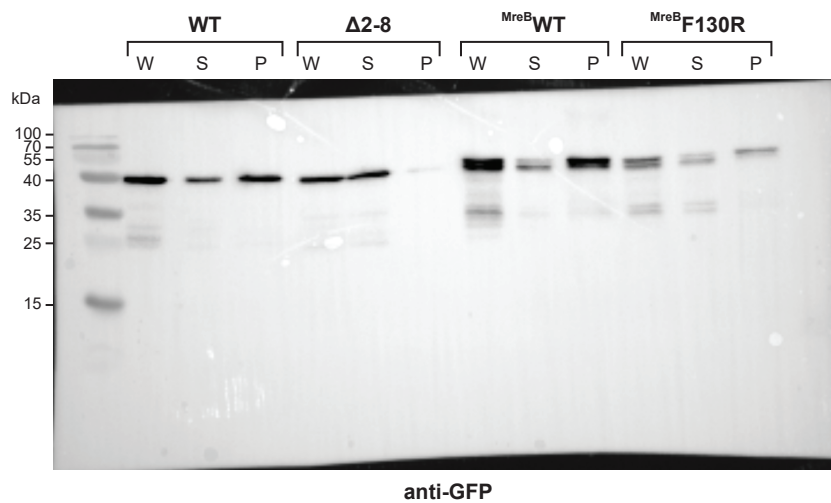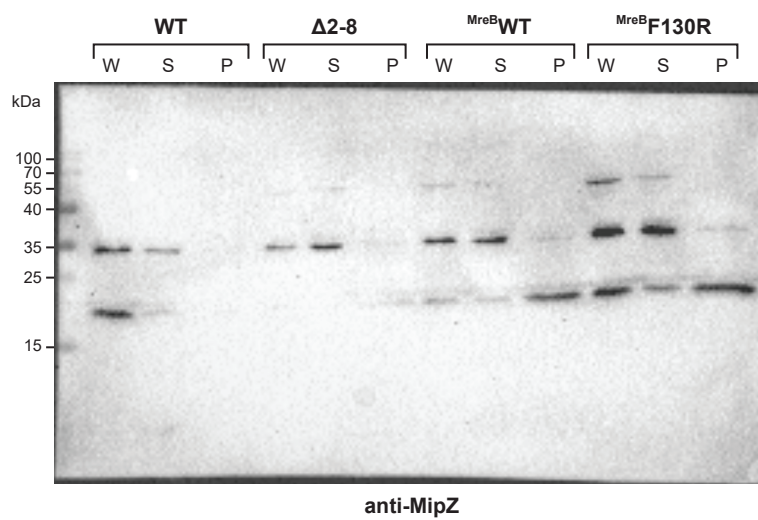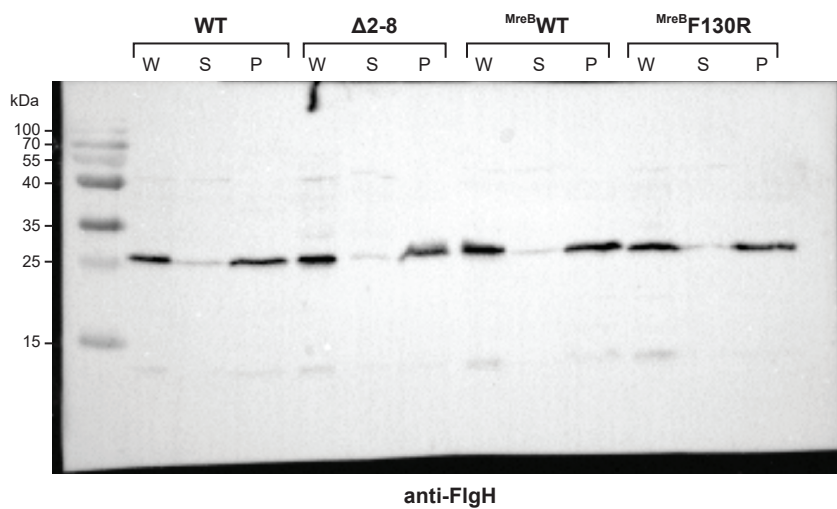

Supplement: Figure 2—source data 1. [file elife-100749-fig2-data1.zip › Figure 2B and Figure 4E-source data.pdf]

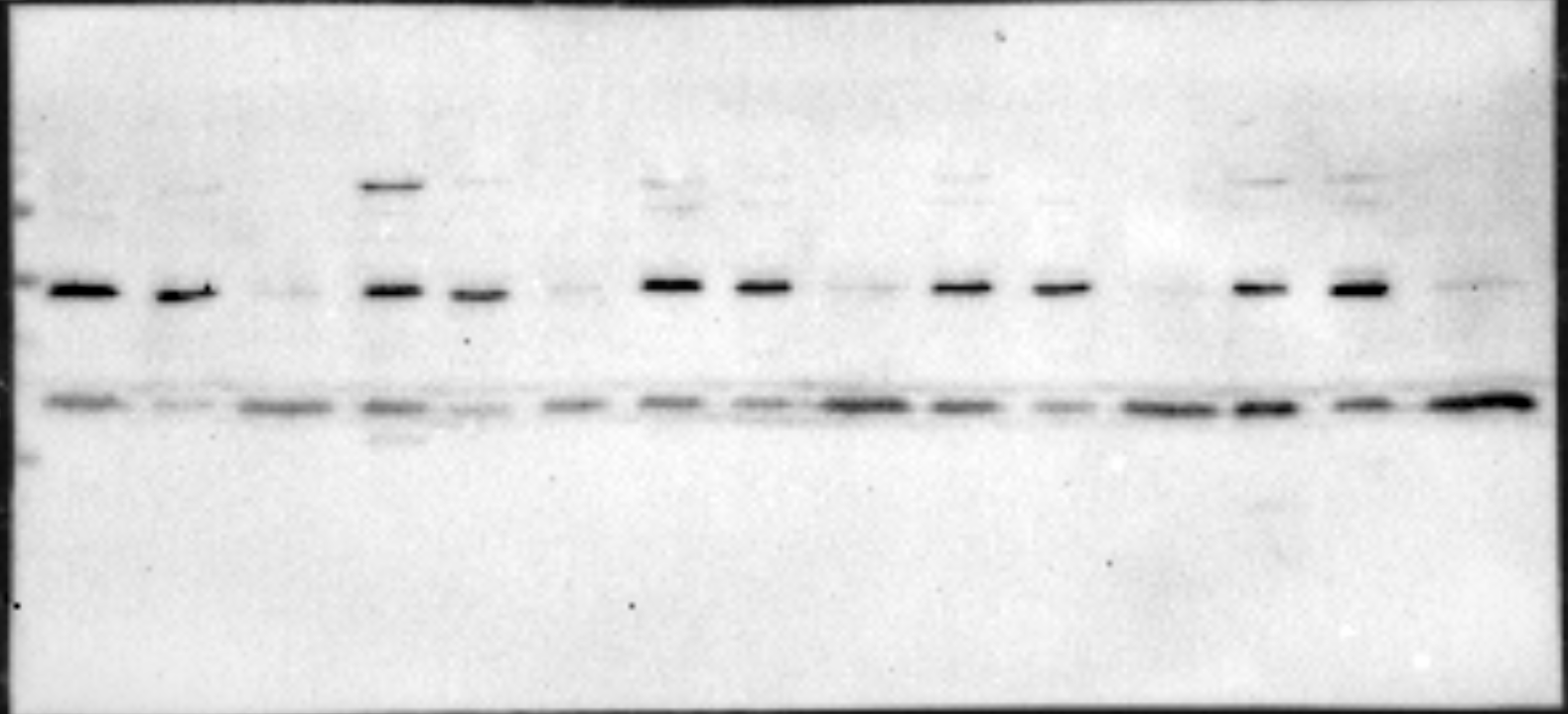

Supplement: Figure 2—source data 2. [file elife-100749-fig2-data2.zip › Figure 2C-source data-anti-MipZ.tif]

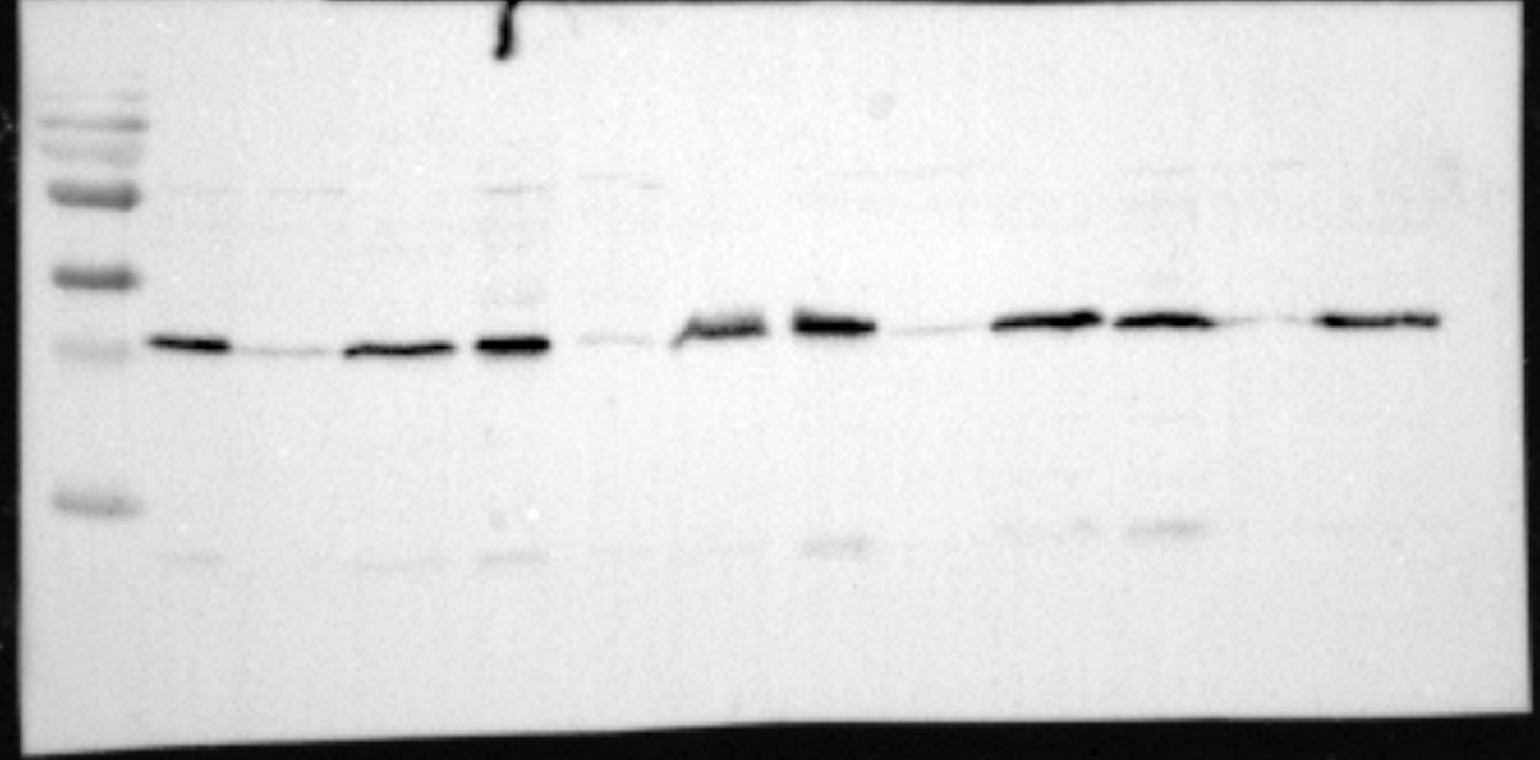

Supplement: Figure 2—source data 2. [file elife-100749-fig2-data2.zip › Figure 2B and Figure 4E-source data-anti-FlgH.tif]

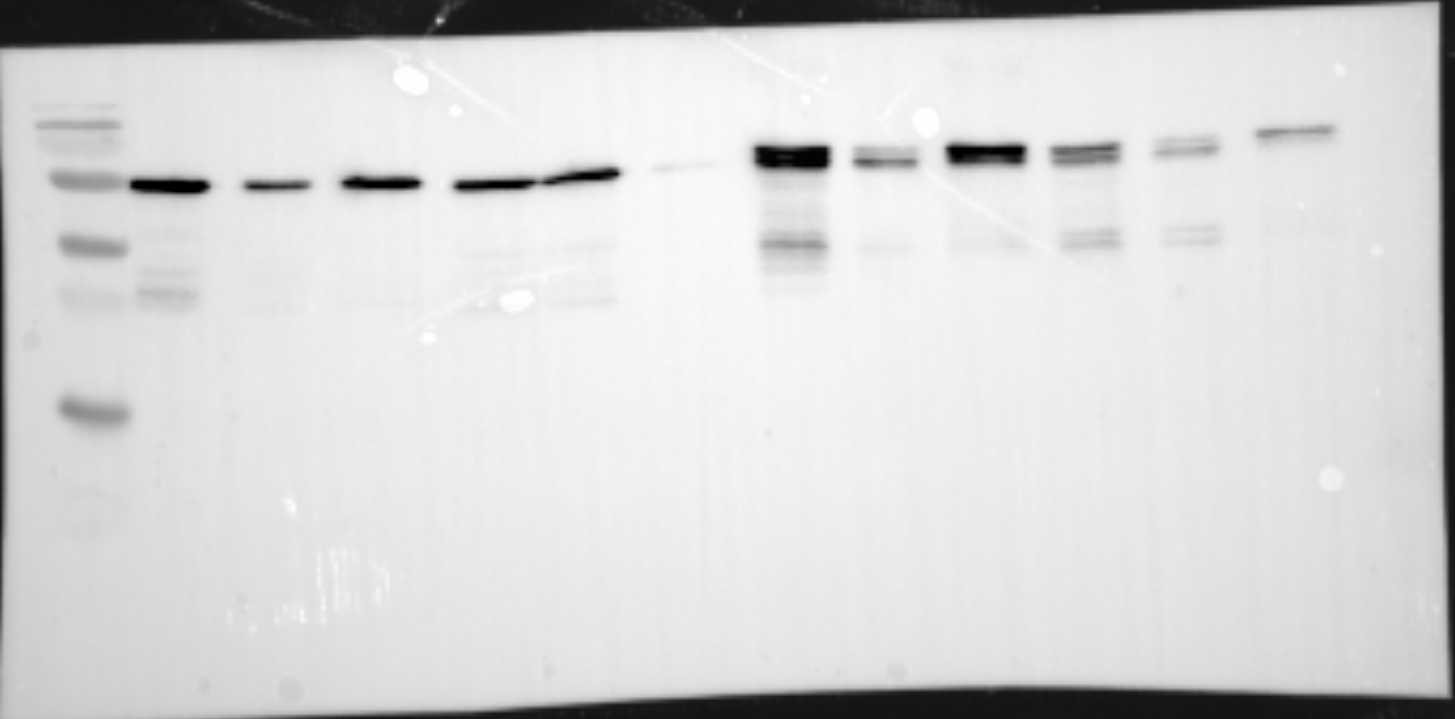

Supplement: Figure 2—source data 2. [file elife-100749-fig2-data2.zip › Figure 2B and Figure 4E-source data-anti-GFP.tif]

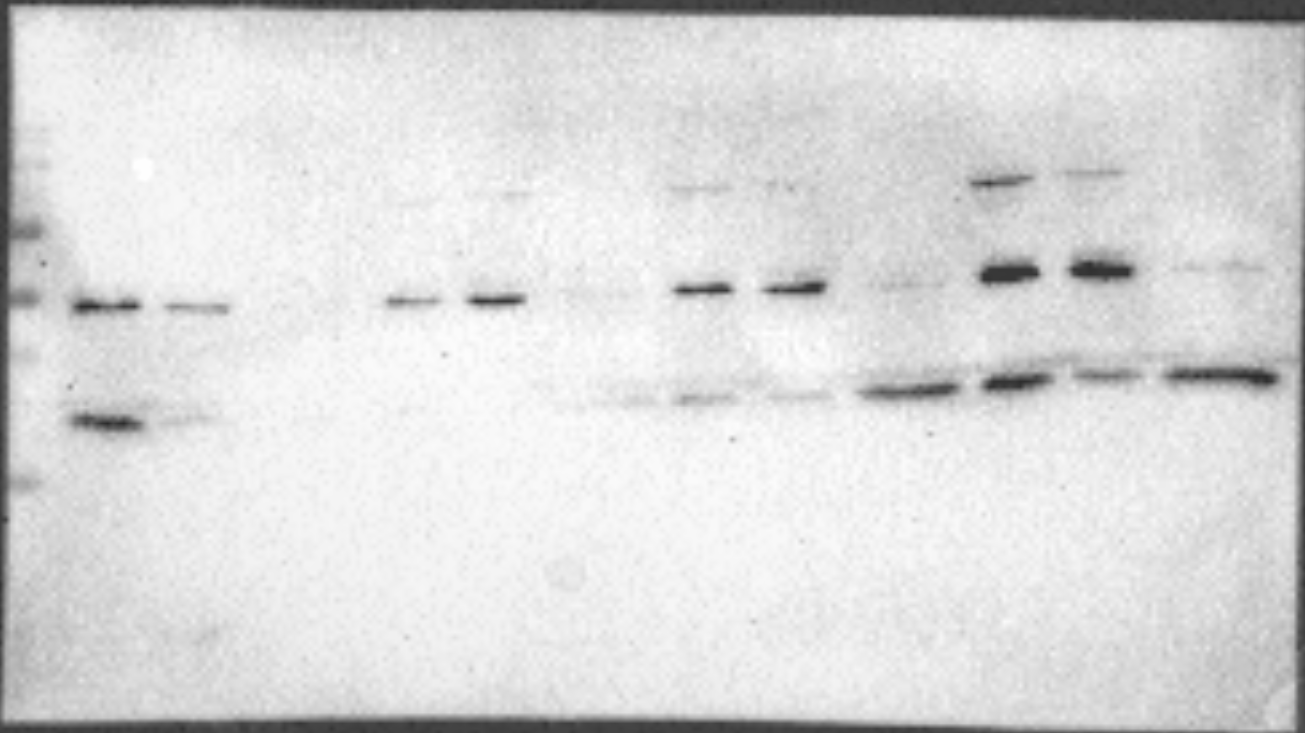

Supplement: Figure 2—source data 2. [file elife-100749-fig2-data2.zip › Figure 2B and Figure 4E-source data-anti-MipZ.tif]

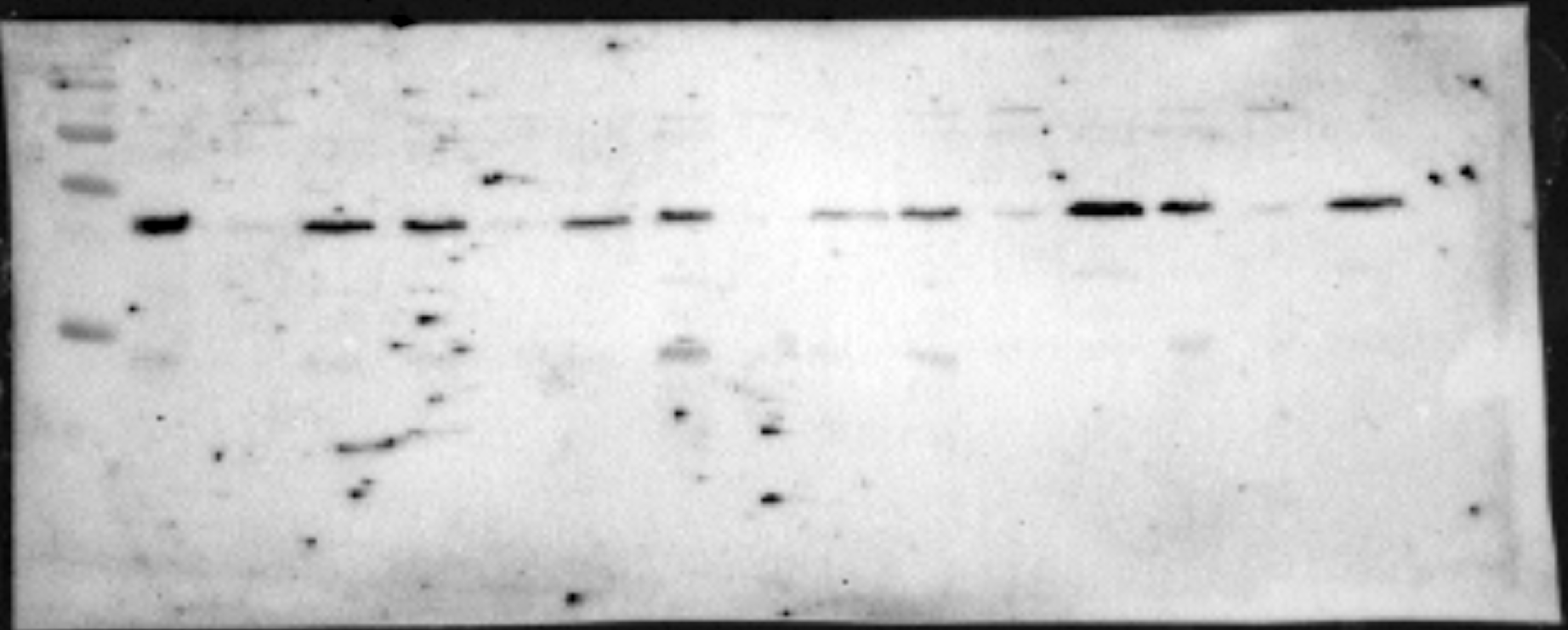

Supplement: Figure 2—source data 2. [file elife-100749-fig2-data2.zip › Figure 2C-source data-anti-FlgH.tif]

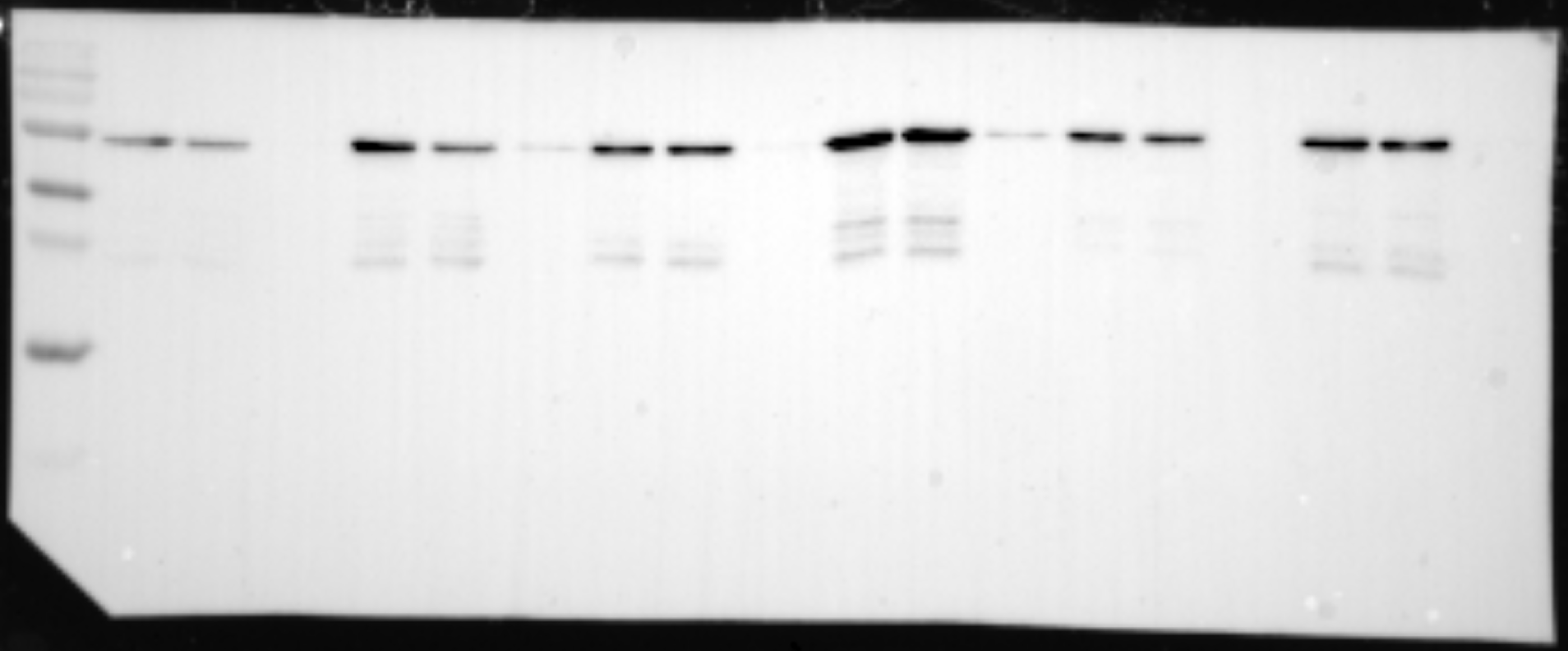

Supplement: Figure 2—source data 2. [file elife-100749-fig2-data2.zip › Figure 2C-source data-anti-GFP.tif]

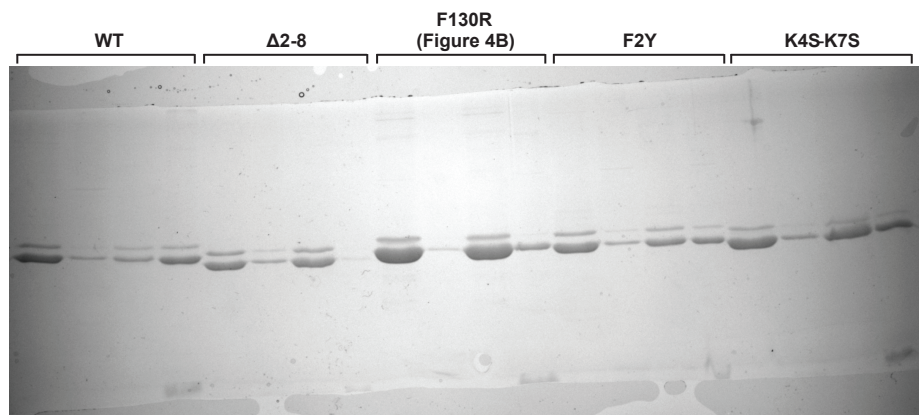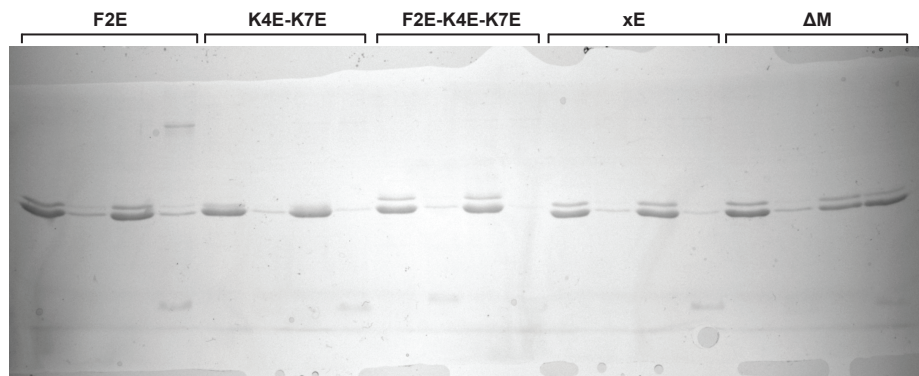

Supplement: Figure 3—source data 1. [file elife-100749-fig3-data1.zip › Figure 3-source data.pdf]

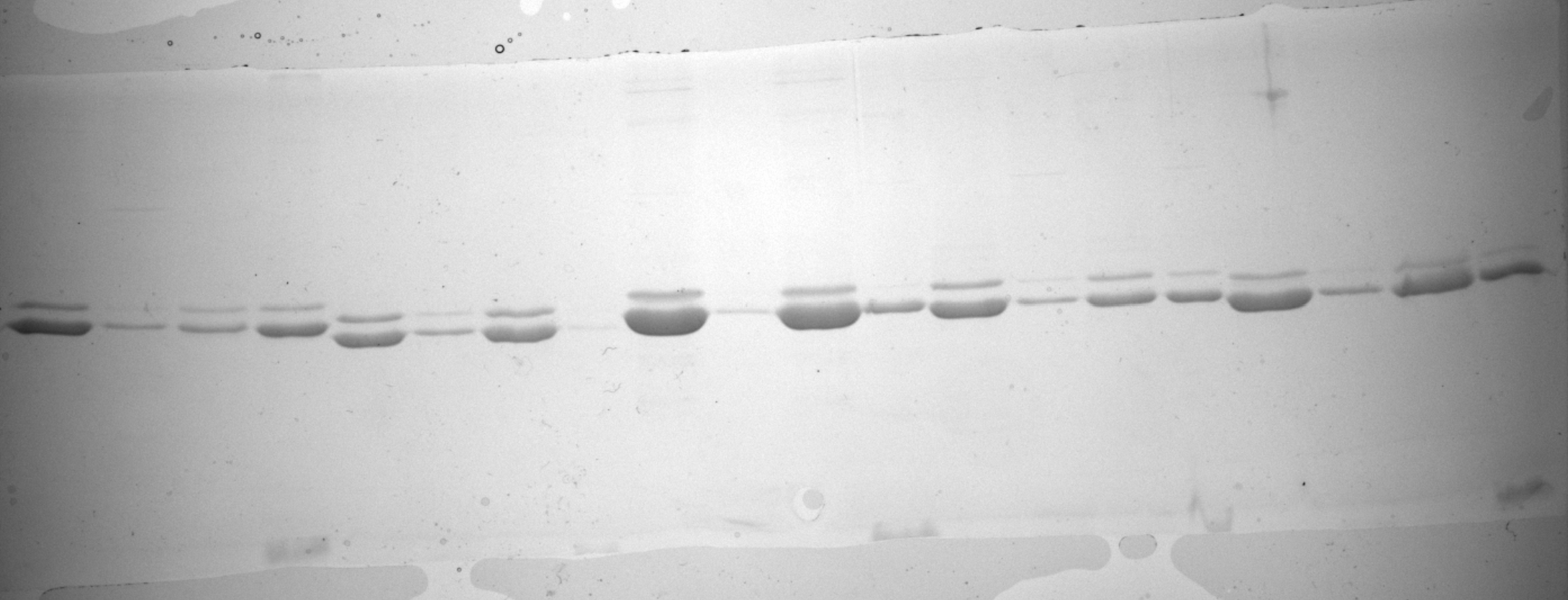

Supplement: Figure 3—source data 2. [file elife-100749-fig3-data2.zip › Figure 3-source data-upper panel.tif]

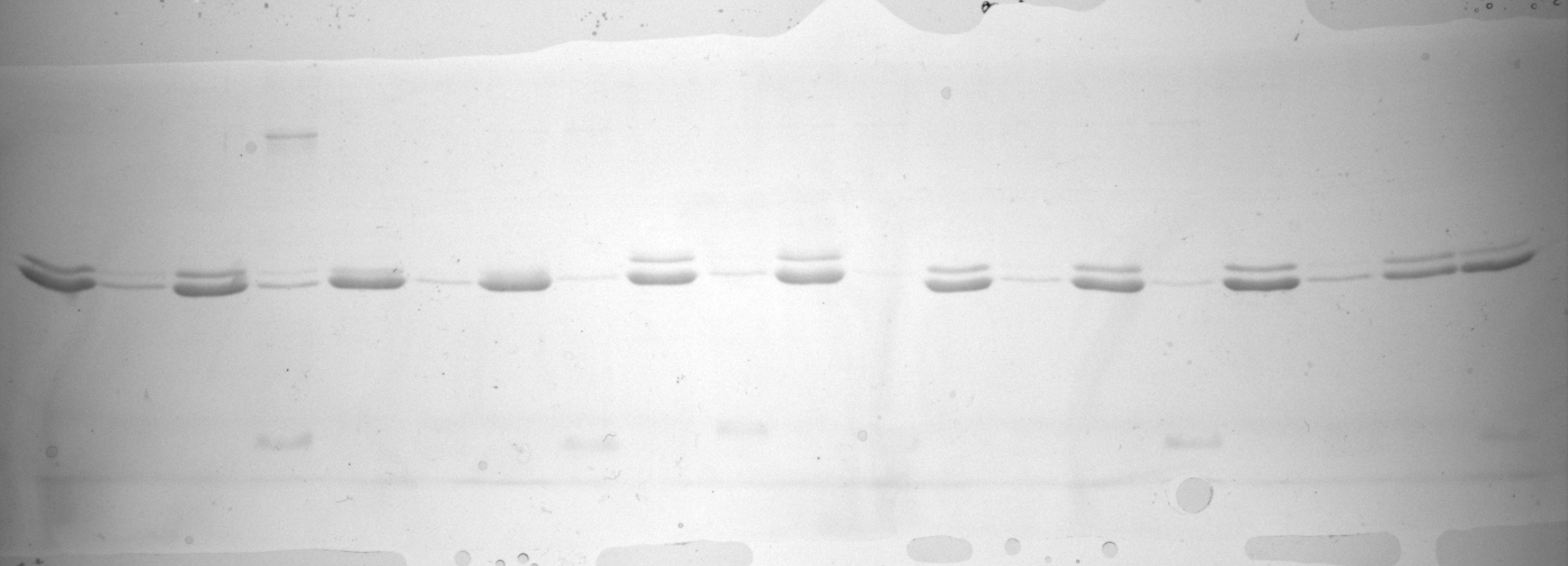

Supplement: Figure 3—source data 2. [file elife-100749-fig3-data2.zip › Figure 3-source data-lower panel.tif]

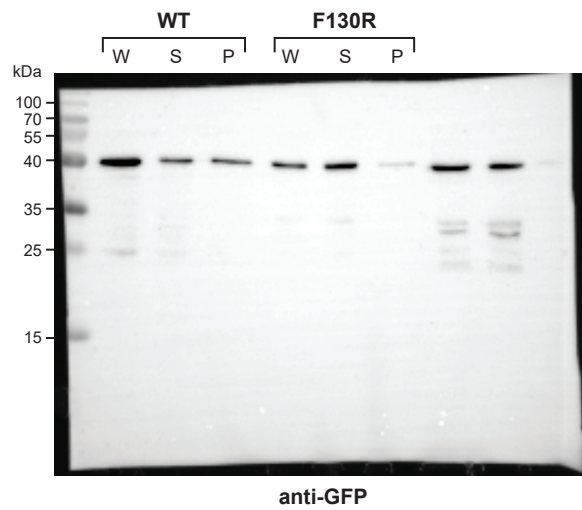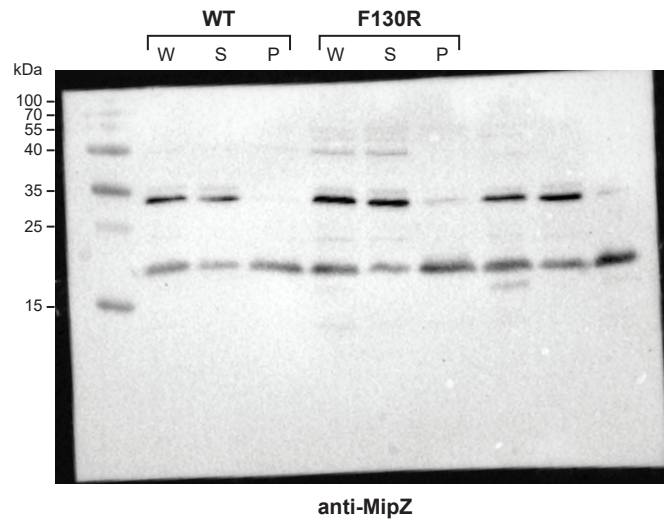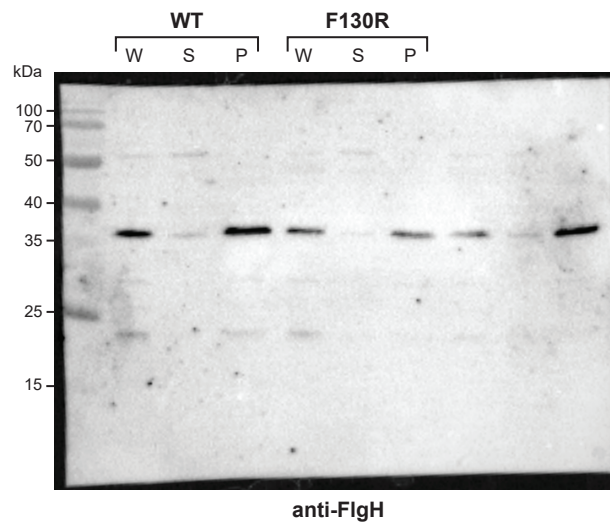

Supplement: Figure 4—source data 1. [file elife-100749-fig4-data1.zip › Figure 4A-source data.pdf]

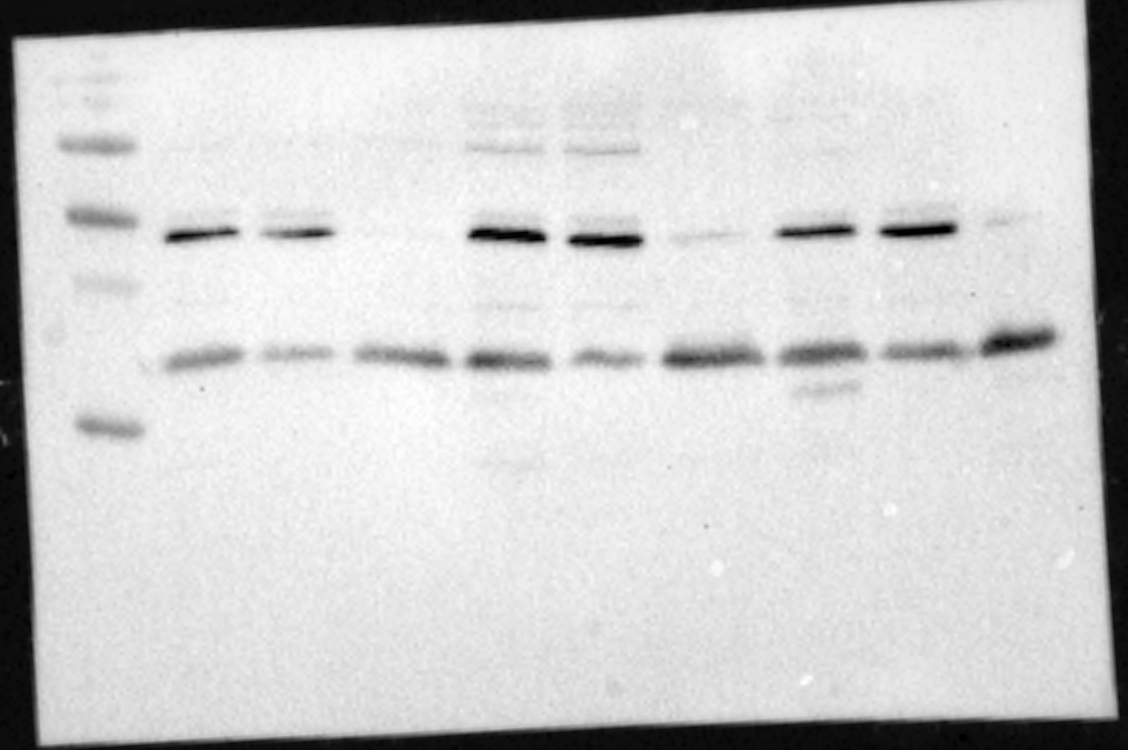

Supplement: Figure 4—source data 2. [file elife-100749-fig4-data2.zip › Figure 4A-source data-anti-MipZ.tif]

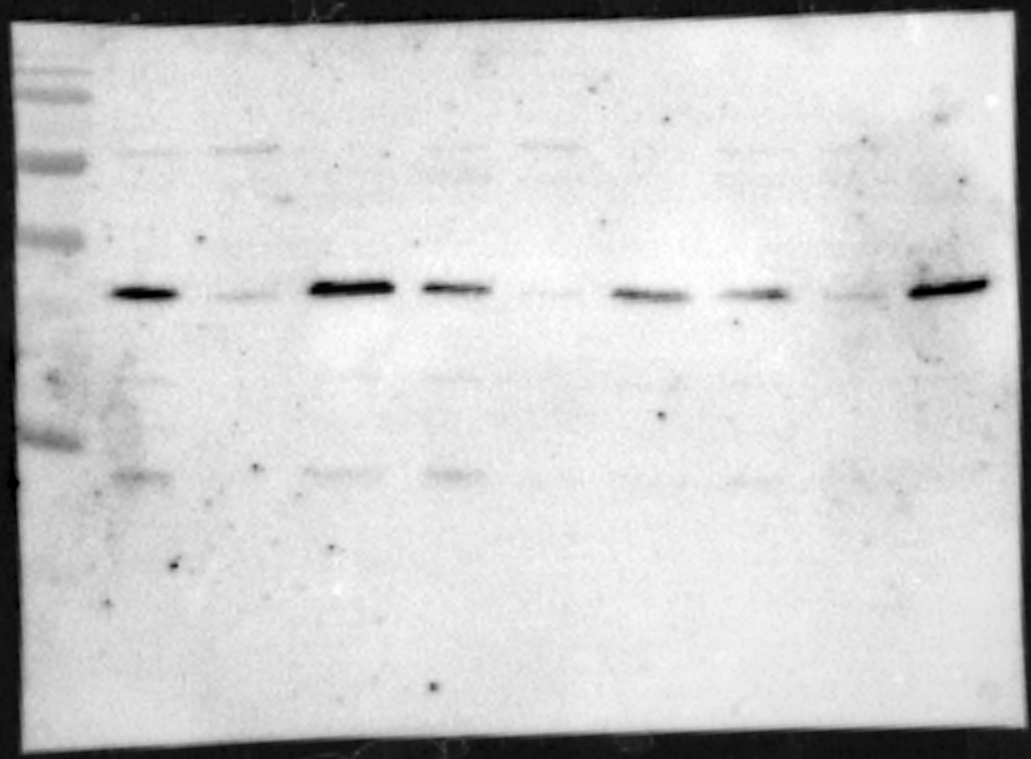

Supplement: Figure 4—source data 2. [file elife-100749-fig4-data2.zip › Figure 4A-source data-anti-FlgH.tif]

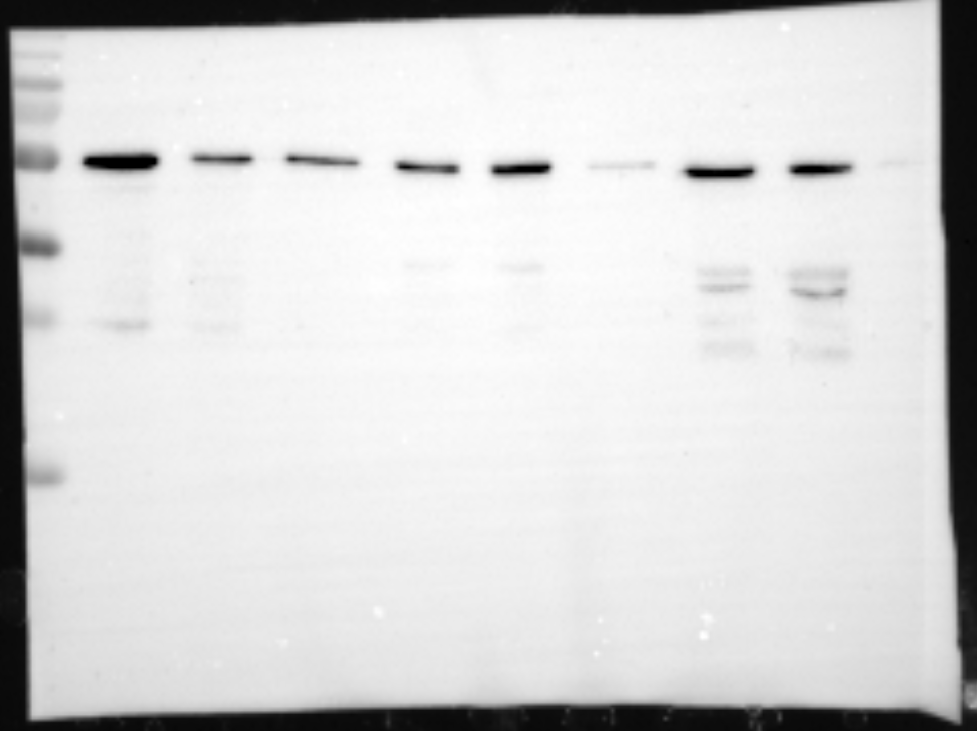

Supplement: Figure 4—source data 2. [file elife-100749-fig4-data2.zip › Figure 4A-source data-anti-GFP.tif]

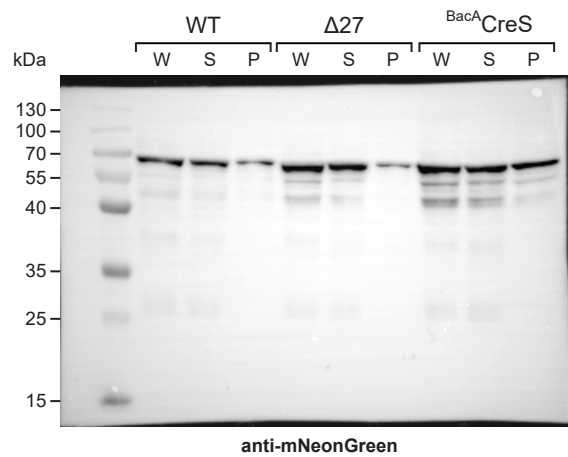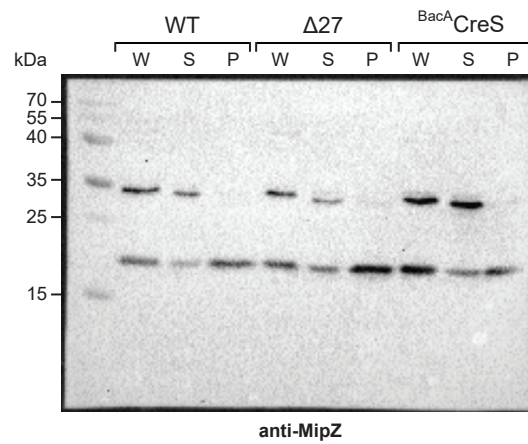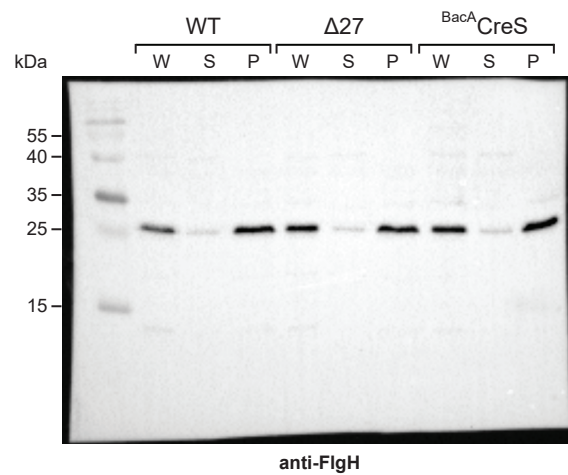

Supplement: Figure 4—figure supplement 1—source data 1. [file elife-100749-fig4-figsupp1-data1.pdf]

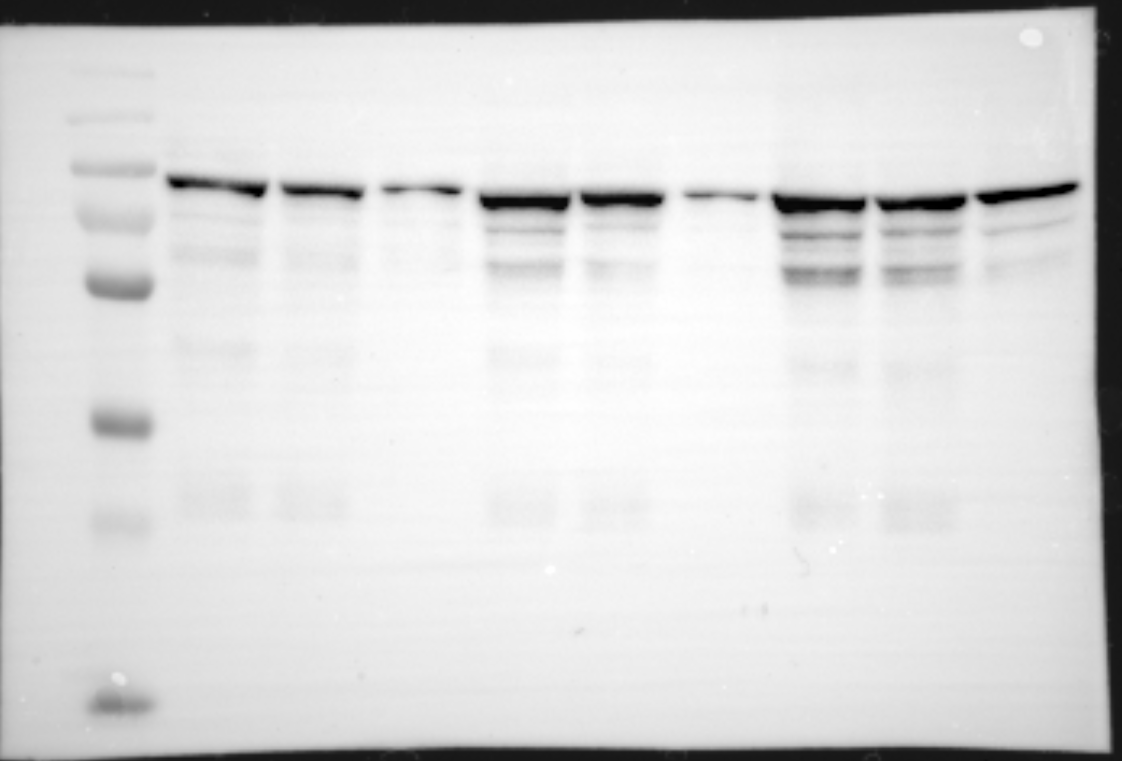

Supplement: Figure 4—figure supplement 1—source data 2. [file elife-100749-fig4-figsupp1-data2.zip › Figure 4-figure supplement 1B-source data-anti-mNeonGreen.tif.tif]

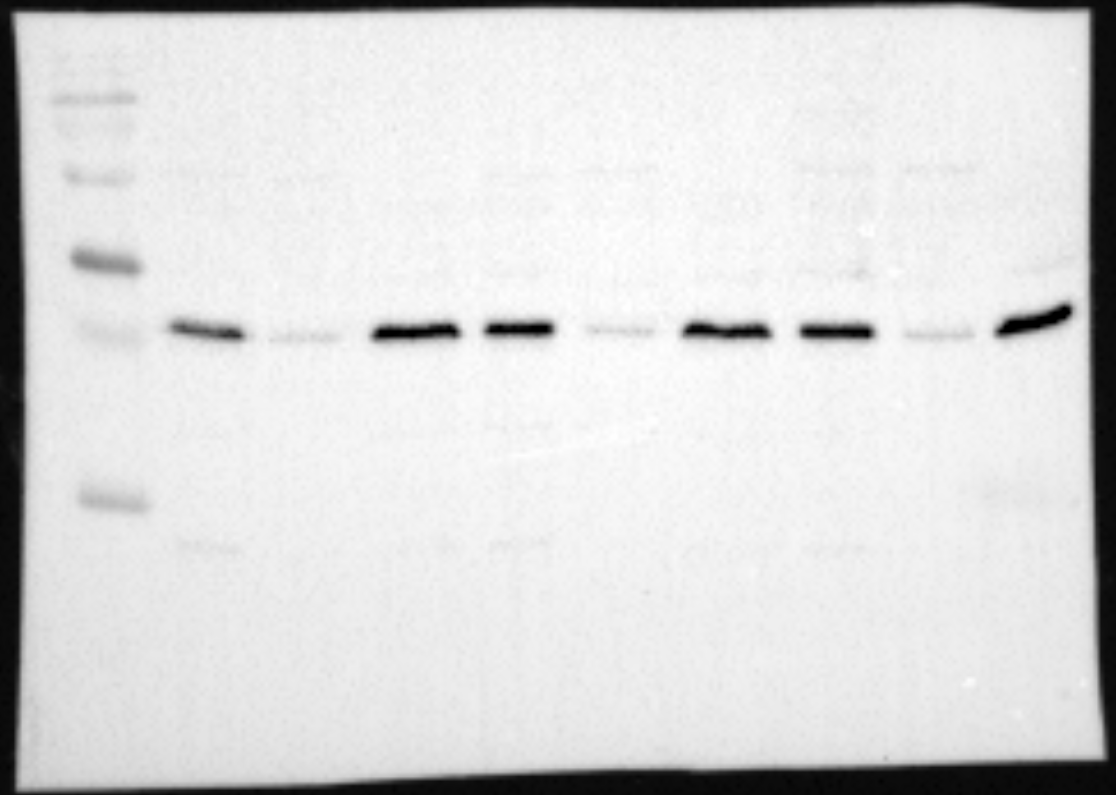

Supplement: Figure 4—figure supplement 1—source data 2. [file elife-100749-fig4-figsupp1-data2.zip › Figure 4-figure supplement 1B-source data-anti-FlgH.tif]

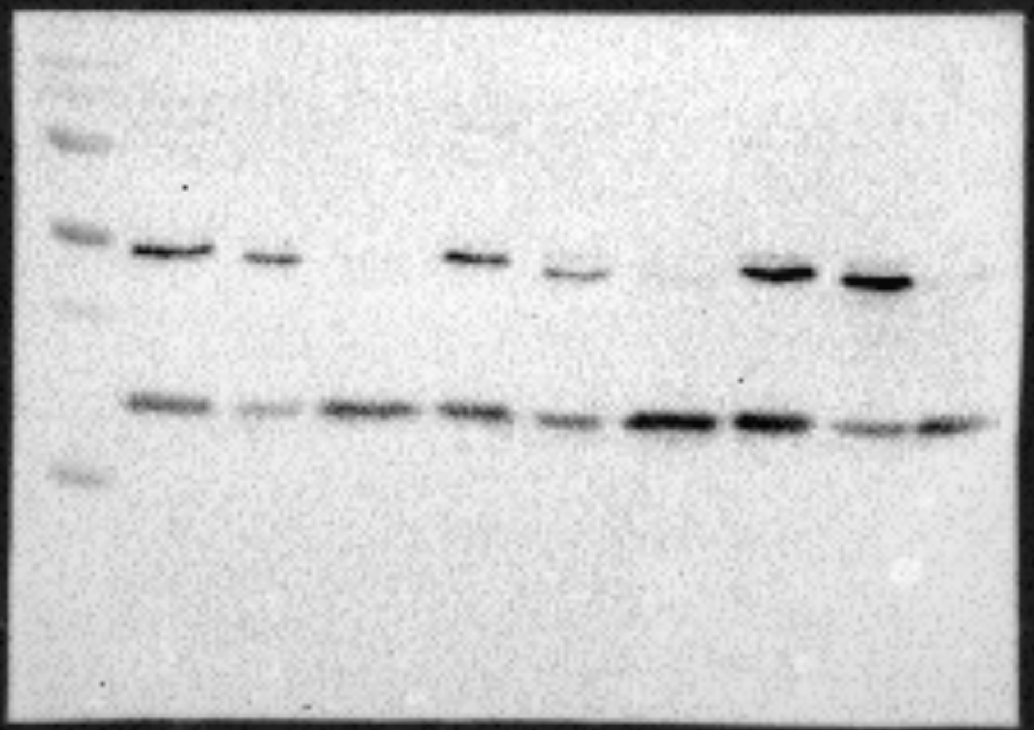

Supplement: Figure 4—figure supplement 1—source data 2. [file elife-100749-fig4-figsupp1-data2.zip › Figure 4-figure supplement 1B-source data-anti-MipZ.tif.tif]

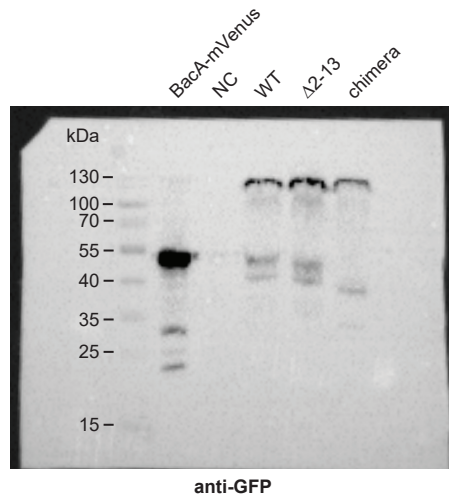

Supplement: Figure 8—figure supplement 3—source data 1. [file elife-100749-fig8-figsupp3-data1.zip › Figure 8-figure supplement 3-source data.pdf]

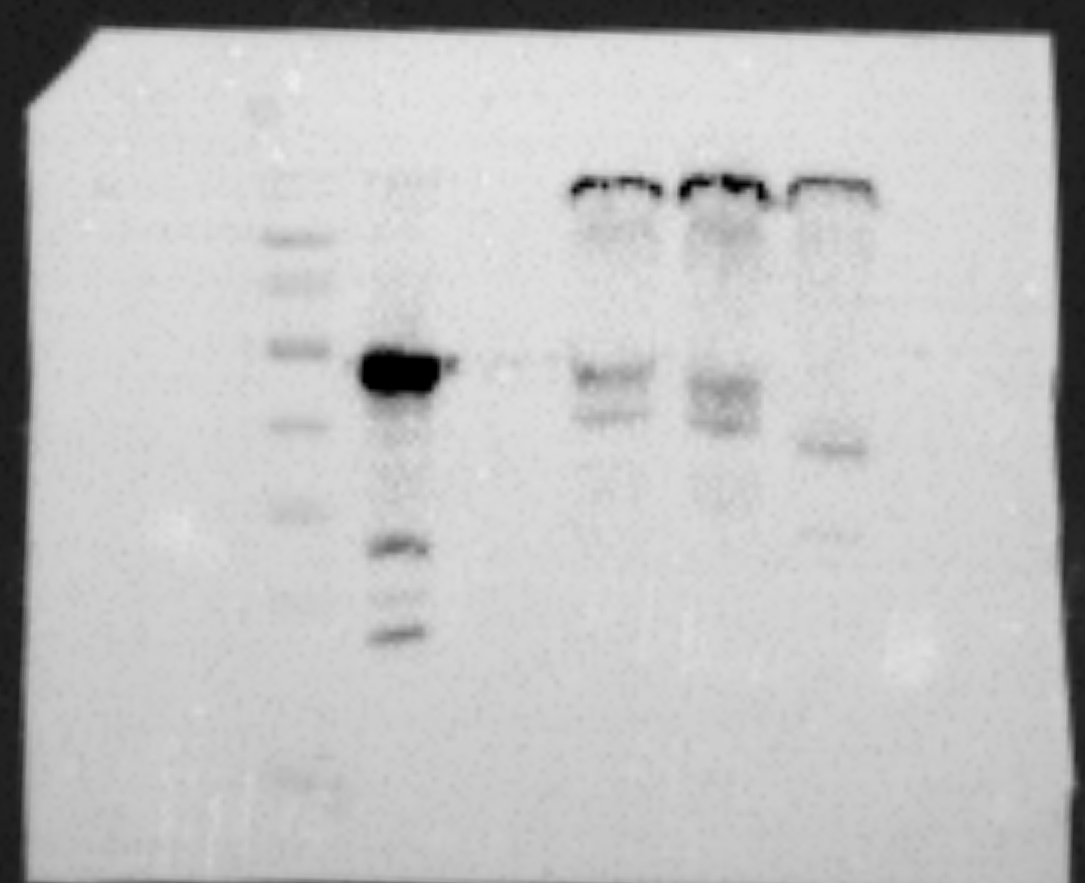

Supplement: Figure 8—figure supplement 3—source data 2. [file elife-100749-fig8-figsupp3-data2.zip › Figure 8-figure supplement 3-source data.tif]

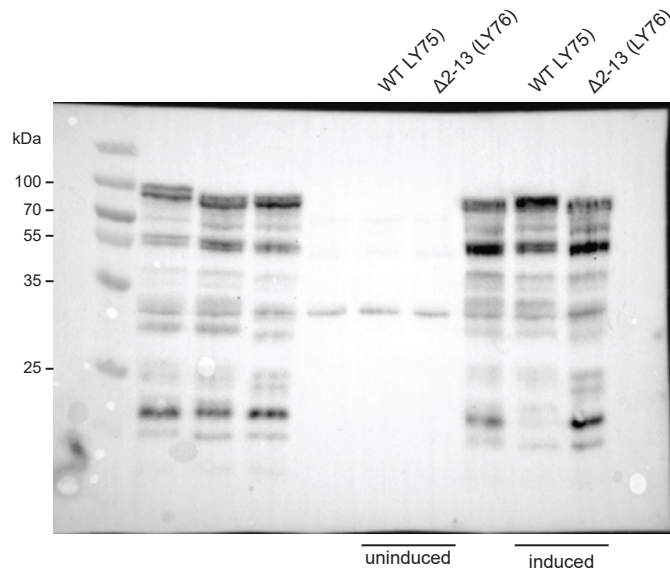

Supplement: Figure 8—figure supplement 4—source data 1. [file elife-100749-fig8-figsupp4-data1.zip › Figure 8-figure supplement 4C-source data.pdf]

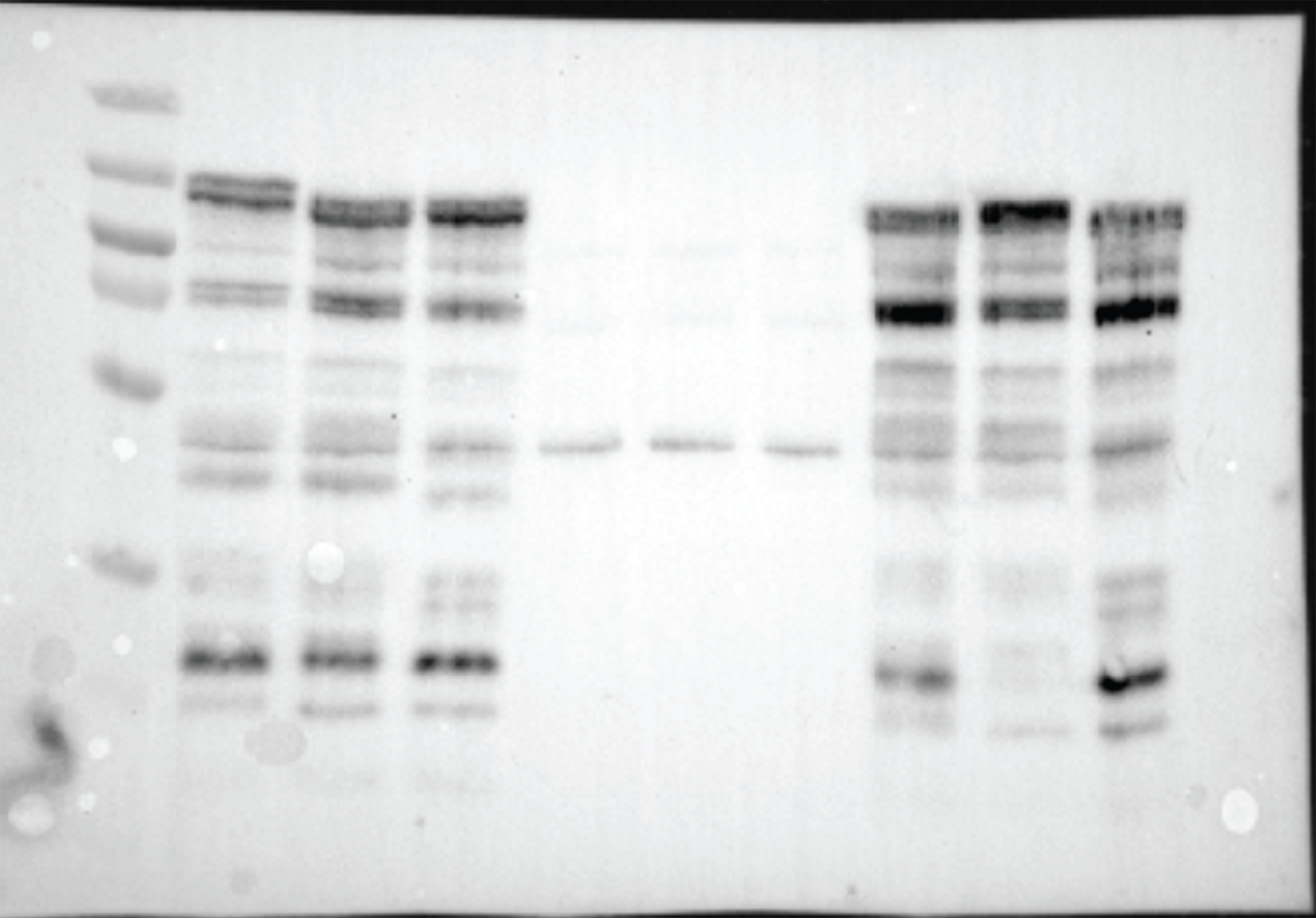

Supplement: Figure 8—figure supplement 4—source data 2. [file elife-100749-fig8-figsupp4-data2.zip › Figure 8-figure supplement 4C-source data.tif]

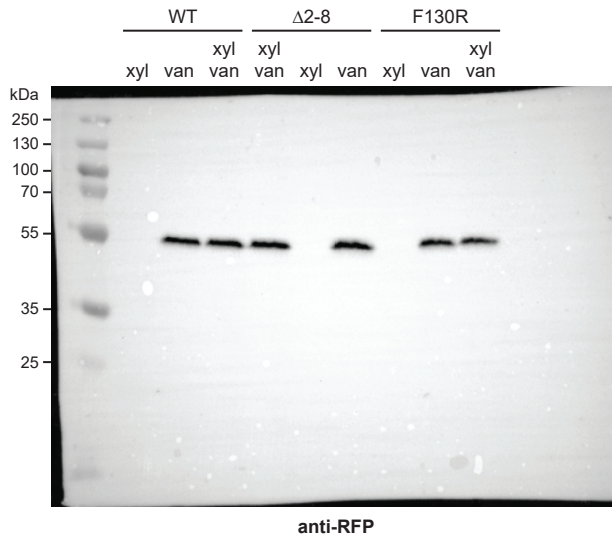

Supplement: Figure 9—figure supplement 1—source data 1. [file elife-100749-fig9-figsupp1-data1.zip › Figure 9-figure supplement 1B-source data.pdf]

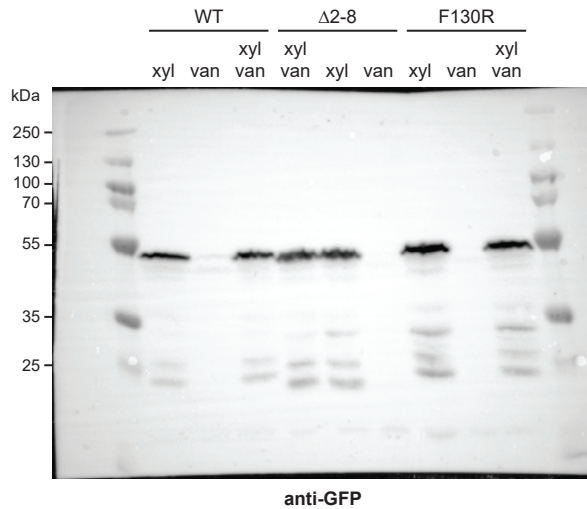

Supplement: Figure 9—figure supplement 1—source data 1. [file elife-100749-fig9-figsupp1-data1.zip › Figure 9-figure supplement 1A-source data.pdf]

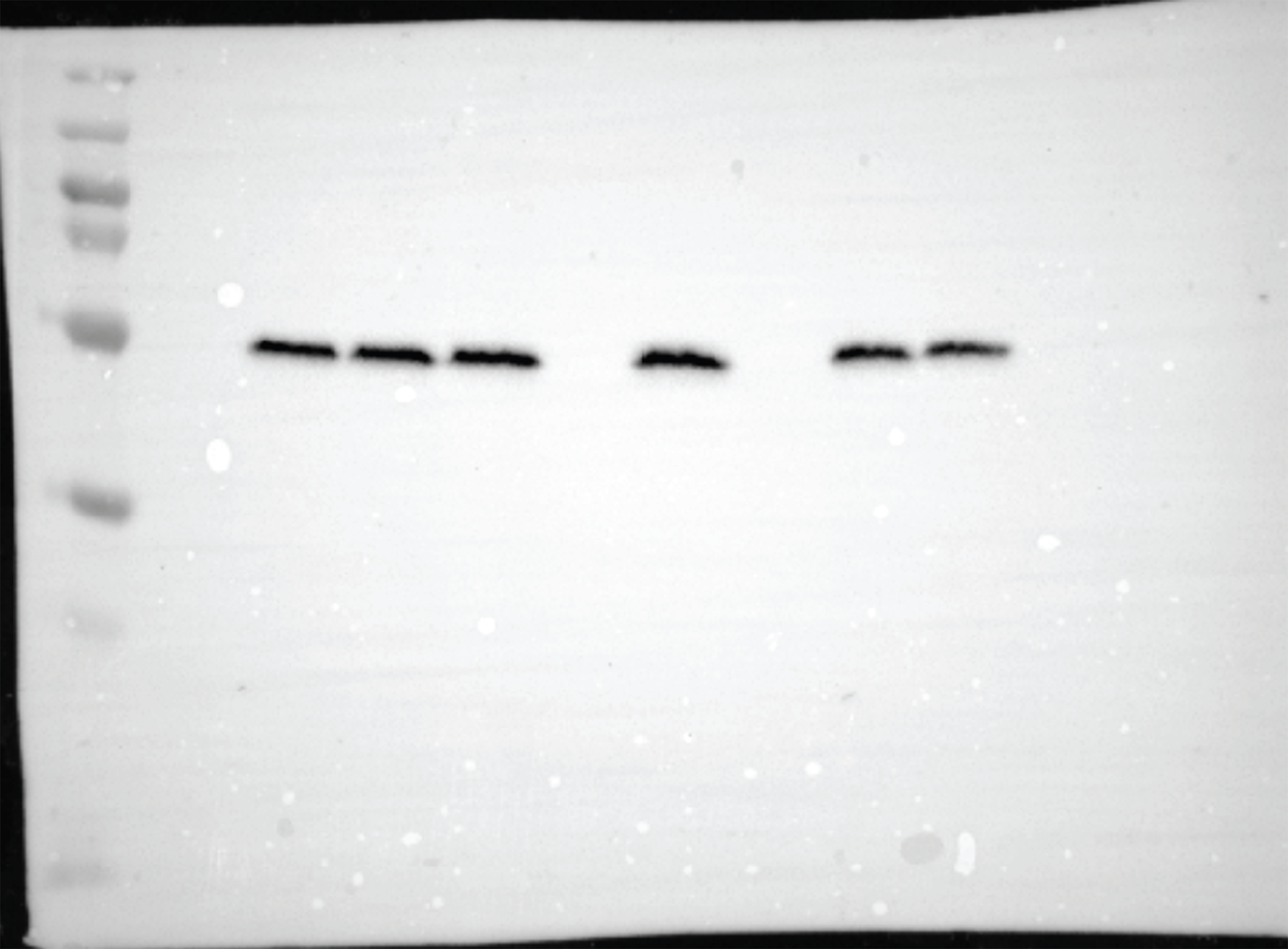

Supplement: Figure 9—figure supplement 1—source data 2. [file elife-100749-fig9-figsupp1-data2.zip › Figure 9-figure supplement 1B-source data.tif]

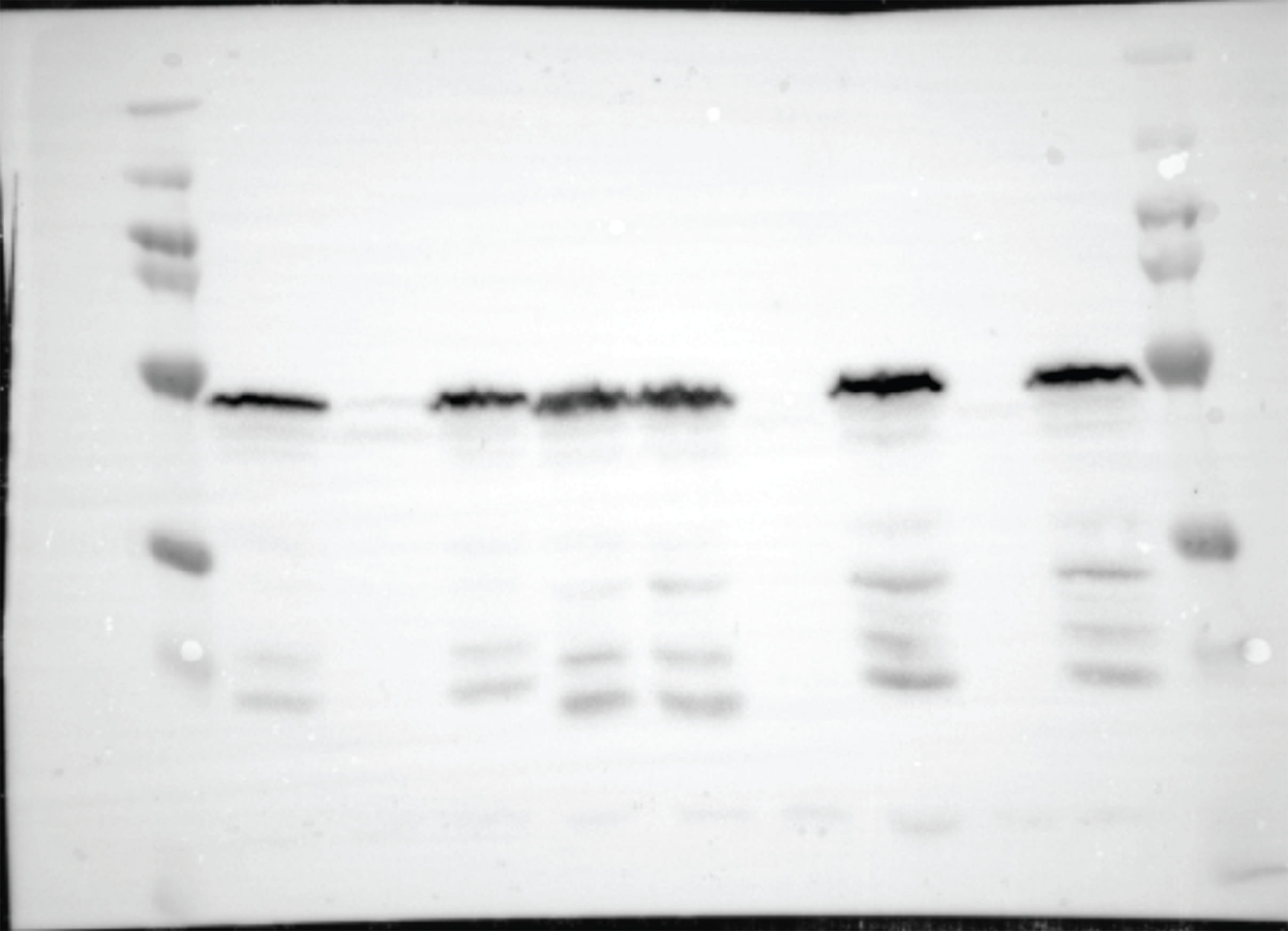

Supplement: Figure 9—figure supplement 1—source data 2. [file elife-100749-fig9-figsupp1-data2.zip › Figure 9-figure supplement 1A-source data.tif]
