# Supplementary material for: Membrane binding properties of the cytoskeletal protein bactofilin
Source: eLife. 2025 Sep 19;13:RP100749. doi: 10.7554/eLife.100749 (PMC12448750; doi:10.7554/eLife.100749)
Supplement: Supplementary file 1. — The table shows the number of cells and tracks analyzed in the single-particle tracking studies as well as the diffusion coefficients obtained for each of the proteins investigated. [file elife-100749-supp1.docx]

**Supplementary file 1.** **Diffusion constants of different BacA-mVenus variants.**

| **Variant** | **No. of cells** | **No. of tracks** | ***D*** | ***D*_1_** | ***D*_2_** |
| --- | --- | --- | --- | --- | --- |
| **WT** | 130 | 2723 | 0.06 ± 0.018 | 0.02 ± 0.001 | 0.27 ± 0.001 |
| **mVenus** | 101 | 1809 | 0.29 ± 0.015 | 0.50 ± 0.002 | 1.10 ± 0.002 |
| **Δ2-8** | 120 | 2039 | 0.18 ± 0.017 | 0.24 ± 0.001 | 0.89 ± 0.001 |
| **F2Y** | 134 | 2614 | 0.13 ± 0.011 | 0.18 ± 0.002 | 0.62 ± 0.002 |
| **K4S-K7S** | 107 | 2350 | 0.08 ± 0.018 | 0.04 ± 0.001 | 0.30 ± 0.001 |
| **F2E** | 140 | 2030 | 0.17 ± 0.012 | 0.19 ± 0.001 | 0.66 ± 0.001 |
| **K4E-K7E** | 132 | 2299 | 0.25 ± 0.017 | 0.31 ± 0.002 | 0.87 ± 0.002 |
| **F2E-K4E-K7E** | 108 | 2388 | 0.16 ± 0.015 | 0.22 ± 0.001 | 0.81 ± 0.001 |
| **F130R** | 133 | 2879 | 0.22 ± 0.011 | 0.39 ± 0.001 | 0.92 ± 0.001 |
| **^MreB^F130R** | 116 | 2478 | 0.26 ± 0.014 | 0.33 ± 0.002 | 0.95 ± 0.002 |
| **^MreB^WT** | 123 | 2100 | 0.03 ± 0.019 | 0.01 ± 0.001 | 0.17 ± 0.001 |
|  |  |  |  |  |  |

*D*, MSD, average diffusion constant of all molecules (µm^2^·s^-1^)

*D*_1_, diffusion constant of the slow fraction (µm^2^·s^-1^)

*D*_2_, diffusion constant of the mobile fraction (µm^2^·s^-1^)
