# Supplementary material for: Membrane binding properties of the cytoskeletal protein bactofilin
Source: eLife. 2025 Sep 19;13:RP100749. doi: 10.7554/eLife.100749 (PMC12448750; doi:10.7554/eLife.100749)
Supplement: Supplementary file 2. — The table shows the type, charge, fatty acid composition, percentage share, and number count of the lipids in each of the two leaflets that constitute the lipid bilayer in the molecular dynamics (MD) simulations. [file elife-100749-supp2.docx]

**Supplementary file 2.** **Composition of the lipid bilayer in the MD simulations.**

| **Name of lipid** | **Charge** | **Fatty acid type** | **Percentage share** | **Number count**  (in each layer) |
| --- | --- | --- | --- | --- |
| **Phosphatidylglycerol (PG)** | | | | |
| DLPG | -1 | 12:0/12:0 | 4.68 | 6 |
| DMPG | -1 | 14:0/14:0 | 0.78 | 1 |
| DPPG | -1 | 16:0/16:0 | 1.56 | 2 |
| PYPG | -1 | 16:0/16:1 | 3.12 | 4 |
| YPPG | -1 | 16:1/16:0 | 3.12 | 4 |
| POPG | -1 | 16:0/18:1 | 1.56 | 2 |
| SOPG | -1 | 18:0/18:1 | 2.34 | 3 |
| DYPG | -1 | 16:1/16:1 | 3.91 | 5 |
| DOPG | -1 | 18:1/18:1 | 11.72 | 15 |
| **PG - total** | | | 32.81 | 42 |
| **Diacylglycerol (DAG) lipids** | | | | |
| DLGL | 0 | 12:0/12:0 | 2.34 | 3 |
| DMGL | 0 | 14:0/14:0 | 0.78 | 1 |
| DPGL | 0 | 16:0/16:0 | 2.34 | 3 |
| DSGL | 0 | 18:0/18:0 | 0.78 | 1 |
| DYGL | 0 | 16:1/16:1 | 3.12 | 4 |
| DOGL | 0 | 18:1/18:1 | 6.25 | 8 |
| **DAG - total** | | | 15.62 | 20 |
| **Monoglucosyldiglyceride (GLY)** | | | | |
| DAG-DL | 0 | 12:0/12:0 | 3.90 | 5 |
| DAG-DP | 0 | 16:0/16:0 | 7.81 | 10 |
| DAG-DY | 0 | 16:1/16:1 | 10.15 | 13 |
| DAG-DO | 0 | 18:1/18:1 | 23.44 | 30 |
| DAG-SO | 0 | 18:0/18:1 | 3.90 | 5 |
| **GLY - total** | | | 49.22 | 63 |
| **Phosphatidic acid (PA)** | | | | |
| DPPA | -1 | 16:0/16:0 | 0.78 | 1 |
| POPA | -1 | 16:0/18:1 | 0.78 | 1 |
| DOPA | -1 | 18:1/18:1 | 0.78 | 1 |
| **PA - total** | | | 2.34 | 3 |
| **TOTAL** | | | 100 | 128 |
