# Supplementary material for: Membrane binding properties of the cytoskeletal protein bactofilin
Source: eLife. 2025 Sep 19;13:RP100749. doi: 10.7554/eLife.100749 (PMC12448750; doi:10.7554/eLife.100749)
Supplement: Supplementary file 5. — The table gives the genotypes, mode of construction and source of all strains used in this study. [file elife-100749-supp5.docx]

**Supplementary file 5.** **Strains used in this study.**

| **Strain** | **Genotype** | **Construction** | **Source** |
| --- | --- | --- | --- |
| ***C. crescentus*** | | | |
| CB15N | Synchronizable variant of wild-type strain CB15 | - | Evinger and Agabian, 1977 |
| JK5 | CB15N ∆*bacAB* | - | Kühn et al., 2010 |
| JK81 | CB15N *creS*::Tn5 | - | Laboratory stock |
| JK136 | CB15N ∆*pbpC xylX*::P*_xyl_-bacA-venus* | - | Kühn et al., 2010 |
| JK281 | Δ*bacAB* Δ*pbpC* |  | Kühn et al., 2010 |
| MT256 | CB15N *xylX*::P*_xyl_-bacA-venus* | - | Laboratory stock |
| MT304 | ∆*pbpC* | - | Kühn et al., 2010 |
| LY70 | ∆*bacA* ∆*pbpC* | In-frame deletion of *bacA* in MT304 using pMT813 | This work |
| LY71 | ∆*bacB* ∆*pbpC* | In-frame deletion of *bacB* in MT304 using pMT815 | This work |
| LY72 | ∆*bacA* ∆*pbpC xylX*::P*_xyl_-mVenus-pbpC* | Integration of pLY073 in LY70 | This work |
| LY75 | ∆*bacB* ∆*pbpC xylX*::P*_xyl_-mVenus-pbpC* | Integration of pLY073 in LY71 | This work |
| LY76 | ∆*bacB* ∆*pbpC xylX*::P*_xyl_-mVenus-pbpC_∆2-13_* | Integration of pLY074 in LY71 | This work |
| LY77 | Δ*bacB* Δ*pbpC xylX*::P*_xyl_-mvenus-pbpC_1-13_-dipM_224-296_-pbpC_84-733_* | Integration of pLY075 in LY71 | This work |
| LY84 | Δ*bacAB xylX*::P*_xyl_-bacA_Δ2-8_-mVenus* | Integration of pLY076 in JK5 | This work |
| LY88 | Δ*bacAB xylX*::P*_xyl_-bacA_K4S_-mVenus* | Integration of pLY087 in JK5 | This work |
| LY89 | Δ*bacAB xylX*::P*_xyl_-bacA_K4S/K7S_-mVenus* | Integration of pLY088 in JK5 | This work |
| LY90 | Δ*bacAB xylX*::P*_xyl_-bacA-mVenus* | Integration of pLY086 in JK5 | This work |
| LY91 | Δ*bacAB xylX*::P*_xyl_-bacA_A6S_-mVenus* | Integration of pLY101 in JK5 | This work |
| LY92 | Δ*bacAB xylX*::P*_xyl_-bacA_K7S_-mVenus* | Integration of pLY102 in JK5 | This work |
| LY95 | Δ*bacAB xylX*::P*_xyl_-bacA_S3A_-mVenus* | Integration of pLY099 in JK5 | This work |
| LY96 | Δ*bacAB xylX*::P*_xyl_-bacA_Q5A_-mVenus* | Integration of pLY100 in JK5 | This work |
| LY97 | Δ*bacAB xylX*::P*_xyl_-bacA_F2Y_-mVenus* | Integration of pLY104 in JK5 | This work |
| LY103 | Δ*bacAB xylX*::P*_xyl_*-2×*mreB_1-11_*-*bacA_Δ2-8_*-*mVenus* | Integration of pLY115 in JK5 | This work |
| LY111 | Δ*bacAB xylX*::P*_xyl_-bacA_F2E_-mVenus* | Integration of pLY131 in JK5 | This work |
| LY112 | Δ*bacAB xylX*::P*_xyl_-bacA_K4E/K7E_-mVenus* | Integration of pLY132 in JK5 | This work |
| LY113 | Δ*bacAB xylX*::P*_xyl_-bacA_F2E/K4E/K7E_-mVenus* | Integration of pLY138 in JK5 | This work |
| LY119 | Δ*bacAB xylX*:: P*_xyl_-bacA_F130R_-mVenus* | Integration of pLY154 in JK5 | This work |
| LY120 | *creS*::Tn5 *xylX*::P*_xyl_-creS-mNeonGreen* | Integration of pLY149 in JK81 | This work |
| LY121 | *creS*::Tn5 *xylX*::P*_xyl_-creS_∆2-27_-mNeonGreen* | Integration of pLY144 in JK81 | This work |
| LY122 | *creS*::Tn5 *xylX*::P*_xyl_-bacA_1-8_-creS_28-457_-mNeonGreen* | Integration of pLY145 in JK81 | This work |
| LY123 | ∆*bacAB* *xylX*::P*_xyl_-*2×*^Ec^mreB_1-11_-bacA_F130R/9-161_-mVenus* | Integration of pLY155 into JK5 | This work |
| MAB568 | Δ*bacAB* Δ*pbpC* *vanA*::P*_van_*-*pbpC_1-132_*-*mCherry* | Integration of pMAB234 into JK281 | This work |
| MAB575 | Δ*bacAB* Δ*pbpC* *vanA*::P*_van_*-*pbpC_1-132_*-*mCherry* *xylX*::P*_van_-bacA_Δ2-8_-mVenus* | Integration of pLY76 into MAB568 | This work |
| MAB576 | Δ*bacAB* Δ*pbpC* *vanA*::P*_van_*-*pbpC_1-132_*-*mCherry* *xylX*::P*_van_-bacA-mVenus* | Integration of pLY86 into MAB568 | This work |
| MAB577 | Δ*bacAB* Δ*pbpC* *vanA*::P*_van_*-*pbpC_1-132_*-*mCherry* *xylX*::P*_van_-bacA_F130R_-mVenus* | Integration of pLY154 into MAB568 | This work |
| ***E. coli*** |  |  |  |
| TOP10 | F^–^ *mcrA* Δ(*mrr-hsdRMS*-*mcrBC*) Φ80*lacZ*ΔM15 Δ*lacX74* *recA1* *araD139* Δ(*ara-leu*)7697 *galU* *galK* *rpsL* (Str^R^) *endA1* *nupG* | - | Invitrogen |
| Rosetta(DE3)pLysS | F^–^ *ompT* *hsdS*_B_(r_B_- m_B_-) *gal dcm* (DE3) pLysSRARE (Cam^R^) | - | Merck Milipore |
