# Supplementary material for: Membrane binding properties of the cytoskeletal protein bactofilin
Source: eLife. 2025 Sep 19;13:RP100749. doi: 10.7554/eLife.100749 (PMC12448750; doi:10.7554/eLife.100749)
Supplement: Supplementary file 6. — The table provides descriptions of all the plasmids used in this study, including details of their construction or source. [file elife-100749-supp6.docx]

**Supplementary file 6.** **Plasmids used in this study.**

| **Plasmid** | **Description** | **Construction/Source** |
| --- | --- | --- |
| pAI039 | Integrative plasmid bearing *dipM*-*sfmTurquoise2^ox^* | Izquierdo-Martinez et al., 2023 |
| pBAD24 | Replicating plasmid for the expression of genes under the control of the arabinose-inducible P_BAD_ promoter, Amp^R^ | Guzman et al., 1995 |
| pET51b(+) | Plasmid for overexpressing proteins with a cleavable N-terminal Strep-II tag and a C-terminal 10xHis tag | Novagen |
| pLY009 | pTB146 bearing *bacA*_F130R_ | (a) Amplification of *bacA*_F130R_ from pLY154 by PCR with primers oLY001 and oLY002  (b) Insertion into *Sap*I/*BamH*I-treated pTB146 by Gibson assembly |
| pLY070 | pXCHYC-2 bearing *pbpC*_1-39nt_*-dipM*_670-888nt_*-pbpC*_250-396nt_ | (a) Amplification of P*_xyl_*-*pbpC*_1-39nt_ from pMT993 by PCR with primers CS008 and oLY158  (b) Amplification of *dipM*_670-888nt_ from pAI039 by PCR with primers oLY159 and oLY160  (c) Amplification of *pbpC*_250-396nt_-*mCherry* from pMT993 by PCR with primers oLY161 and oLY162  (d) Gibson assembly of the three fragments  (e) Digestion of pXCHYC-2 and the assembly product with *Asc*I and *Nhe*I and subsequent ligation |
| pLY073 | pXmVENN-1 bearing *pbpC* | (a) Digestion of pMT906 with *Nhe*I and *Kpn*I  (b) Ligation of the released *pbpC* fragment into *Nhe*I/*Kpn*I-treated pXmVENN-1 |
| pLY074 | pXmVENN-1 bearing *pbpC*_∆4-39nt_ | (a) Amplification of *pbpC*_∆4-39nt_ from pMT906 by PCR with primers oLY169 and CC3277-rev2  (b) Restriction of the PCR product with *Nhe*I and *Kpn*I  (c) Ligation into *Nhe*I/*Kpn*I-treated pXmVENN-1 |
| pLY075 | pXmVENN-1 bearing *pbpC*_1-39nt_*-dipM*_670-888nt_*-pbpC*_250-2202nt_ | (a) Amplification of *pbpC*_1-39nt_*-dipM*_670-888nt_ from pLY070 by PCR with primers CC3277-for and oLY170  (b) Amplification of *pbpC*_250-2202nt_ from pMT906 by PCR with primers oLY171 and CC3277-rev2  (c) Gibson assembly of the two fragments  (d) Digestion of pXmVENN-1 and the assembly product with *Nhe*I and *Kpn*I and subsequent ligation |
| pLY076 | pXmVENC-2 bearing *bacA*_∆4-32nt_ | (a) Amplification of *bacA*_∆4-32nt_ from pMT812 by PCR with primers oLY172 and CC1873-rev  (b) Digestion with *Nde*I and *Sac*I  (c) Ligation into *Nde*I/*Sac*I-treated pXmVENC-2 |
| pLY086 | pXmVENC-2 bearing *bacA* | (a) Digestion of pMT812 with *Nde*I and *Sac*I  (b) Ligation of the released *bacA* fragment into *Nde*I/*Sac*I-treated pXmVENC-2 |
| pLY087 | pXmVENC-2 bearing *bacA*_K4S_ | Site-directed mutagenesis of pLY086 by PCR with primers oLY192 and oLY193 |
| pLY088 | pXmVENC-2 bearing *bacA*_K4S/K7S_ | Site-directed mutagenesis of pLY087 by PCR with primers oLY194 and oLY195 |
| pLY099 | pXmVENC-2 bearing *bacA*_S3A_ | (a) Amplification of *bacA*_S3A_ from a custom-synthesized gene block by PCR with primers oLY217 and oLY218  (b) Amplification of *bacA*_106-483nt_ from pLY086 by PCR with primers oLY219 and CC1873-rev  (c) Gibson assembly of the two fragments  (d) Digestion of pXmVENC-2 and the assembly product with *Nde*I and *Sac*I and subsequent ligation |
| pLY100 | pXmVENC-2 bearing *bacA*_Q5A_ | (a) Amplification of *bacA*_Q5A_ from a custom-synthesized gene block by PCR with primers oLY217 and oLY218  (b) Amplification of *bacA*_106-483nt_ from pLY086 by PCR with primers oLY219 and CC1873-rev  (c) Gibson assembly of the two fragments  (d) Digestion of pXmVENC-2 and the assembly product with *Nde*I and *Sac*I and subsequent ligation |
| pLY101 | pXmVENC-2 bearing *bacA*_A6S_ | (a) Amplification of *bacA*_A6S_ from a custom-synthesized gene block by PCR with primers oLY217 and oLY218  (b) Amplification of *bacA*_106-483nt_ from pLY086 by PCR with primers oLY219 and CC1873-rev  (c) Gibson assembly of the two fragments  (d) Digestion of pXmVENC-2 and the assembly product with *Nde*I and *Sac*I and subsequent ligation |

**Supplementary file 6.** **Plasmids used in this study (continued).**

| **Plasmid** | **Description** | **Construction/Source** |
| --- | --- | --- |
| pLY102 | pXmVENC-2 bearing *bacA*_K7S_ | (a) Amplification of *bacA*_K7S_ from a custom-synthesized gene block by PCR with primers oLY217 and oLY218  (b) Amplification of *bacA*_106-483nt_ from pLY086 by PCR with primers oLY219 and CC1873-rev  (c) Gibson assembly of the two fragments  (d) Digestion of pXmVENC-2 and the assembly product with *Nde*I and *Sac*I and subsequent ligation |
| pLY104 | pXmVENC-2 bearing *bacA*_F2Y_ | Site-directed mutagenesis of pLY086 by PCR with primers oLY222 and oLY223 |
| pLY107 | pET51b(+) bearing MCS-*mVenus* from pXmVENC-2 | (a) Amplification of MCS-*mVenus* from pXmVENC-2 by PCR with primers oLY227 and oLY228  (b) Insertion into *Nco*I/*Avr*II-treated pET51b(+) by Gibson assembly |
| pLY112 | pLY107 bearing 2x*mreB*_EC 1-33nt_ | (a) Annealing of oligonucleotides oLY240 and oLY241  (b) Ligation into pLY107 cut with *Nde*I and *Kpn*I  (c) Annealing of oligonucleotides oLY242 and oLY243  (d) Ligation into the plasmid from step (b) cut with *Xho*I and *EcoR*I |
| pLY115 | pXmVENC-2 bearing 2x*mreB*_EC 1-33nt_-*bacA*_Δ4-32nt_-*mVenus* | (a) Amplification of 2x*mreB*_EC 1-33nt_ from pLY112 by PCR with primers oLY249 and oLY250  (b) Insertion into *Nde*I-treated pLY076 by Gibson assembly |
| pLY116 | pTB146 bearing *bacA*_∆4-32nt_ | (a) Amplification of *bacA*_∆4-32nt_ from pLY076 by PCR with primers oLY251 and oLY002  (b) Insertion into *Sap*I/*BamH*I-treated pTB146 by Gibson assembly |
| pLY117 | pTB146 bearing *bacA*_F2Y_ | (a) Amplification of *bacA*_F2Y_ from pLY104 by PCR with primers oLY252 and oLY002  (b) Insertion into *Sap*I/*BamH*I-treated pTB146 by Gibson assembly |
| pLY118 | pTB146 bearing *bacA*_K4SK7S_ | Amplification of *bacA*_K4SK7S_ from pLY088 by PCR with primers oLY253 and oLY002.  (b) Insertion into *Sap*I/*BamH*I-treated pTB146 by Gibson assembly |
| pLY119 | pTB146 bearing *bacA* | (a) Amplification of *bacA* from pMT812 by PCR with primers oLY001 and oLY002  (b) Insertion into *Sap*I/*BamH*I-treated pTB146 by Gibson assembly |
| pLY131 | pXmVENC-2 bearing *bacA*_F2E_ | Site-directed mutagenesis of pLY086 by PCR with primers oLY269 and oLY270 |
| pLY132 | pXmVENC-2 bearing *bacA*_K4E/K7E_ | (a) Site-directed mutagenesis of in pLY086 by PCR with primers oLY277 and oLY278  (b) Site-directed mutagenesis of the resulting plasmid by PCR with primers oLY279 and oLY280 |
| pLY133 | pTB146 bearing *bacA*_F2E_ | (a) Amplification of *bacA*_F2E_ from pLY131 by PCR with primers oLY273 and oLY002  (b) Insertion into *Sap*I/*BamH*I-treated pTB146 by Gibson assembly. |
| pLY134 | pTB146 bearing *bacA*_xE_ | (a) Amplification of *bacA*_xE_ from pLY131 by PCR with primers oLY274 and oLY002  (b) Insertion into *Sap*I/*BamH*I-treated pTB146 by Gibson assembly |
| pLY135 | pTB146 bearing *bacA*_∆M_ | (a) Amplification of *bacA*_∆M_ from pLY086 by PCR with primers oLY275 and oLY002.  (b) Insertion into *Sap*I/*BamH*I-treated pTB146 by Gibson assembly |
| pLY136 | pTB146 bearing *bacA*_K4E/K7E_ | (a) Amplification of *bacA*_K4EK7E_ from pLY132 by PCR with primers oLY276 and oLY002  (b) Insertion into *Sap*I/*BamH*I-treated pTB146 by Gibson assembly |
| pLY138 | pXmVENC-2 bearing *bacA*_F2E/K4E/K7E_ | (a) Site-directed mutagenesis of pLY131 by PCR with primers oLY281 and oLY278  (b) Site-directed mutagenesis of the resulting plasmid by PCR with primers oLY279 and oLY282 |
| pLY139 | pTB146 bearing *bacA*_F2E/K4E/K7E_ | (a) Amplification of *bacA*_F2EK4EK7E_ from pLY138 by PCR with primers oLY283 and oLY002  (b) Insertion into *Sap*I/*BamH*I-treated pTB146 by Gibson assembly |
| pLY144 | pXmNeonGreenC-4 bearing *creS*_∆1-81nt_ | Deletion of nt 1-81 of *creS* in pLY149 by inverse PCR with primers oLY287 and oLY288 |
| pLY145 | pXmNeonGreenC-4 bearing *bacA*_1-24nt_*-creS*_82-1371nt_ | Replacement of the first 81 nucleotides of *creS* with *bacA*_1-24nt_ in pLY149 by inverse PCR with primers oLY289 and oLY290 |
| pLY149 | pXmNeonGreenC-4 bearing *creS* | (a) Amplification of *creS* from genomic DNA of CB15N by PCR with primers creS-F and oLY301  (b) restriction with *Nde*I and *Kpn*I  (c) Ligation with pXmNeonGreenC-4 cut with *Nde*I and *Kpn*I |
| pLY154 | pXmVENC-2 bearing *bacA*_F130R_ | Site-directed mutagenesis of pLY086 by PCR with primers oLY004 and oLY005 |
| pLY155 | pXmVENC-2 bearing 2x*mreB*_Ec1-33nt_*-bacA* _F130R/31-483nt_ | Site-directed mutagenesis of pLY115 by PCR with primers oLY004 and oLY005 |
|  |  |  |

**Supplementary file 6.** **Plasmids used in this study (continued).**

| **Plasmid** | **Description** | **Construction/Source** |
| --- | --- | --- |
| pMAB234 | pVGFPC-4 bearing pbpC_11-396nt_-*mCherry* | a) Digestion of pMT993 with *Nde*I and *Nhe*I to isolate a fragment containing *pbpC_1_*_1-396nt_-*mCherry*  b) Ligation into pVGFPC-4 cut with *Nde*I and *Nhe*I |
| pMT812 | pXVENC-2 bearing *bacA* | Kühn et al., 2010 |
| pMT813 | pNPTS138 derivative used to generate an in-frame deletion in *bacA* | Kühn et al., 2010 |
| pMT815 | pNPTS138 derivative used to generate an in-frame deletion in *bacB* | Kühn et al., 2010 |
| pMT906 | pXVENN-1 bearing *pbpC* | Kühn et al., 2010 |
| pMT993 | pXCHYC-2 bearing *pbpC*_1-396nt_ | Kühn et al., 2010 |
| pTB146 | Plasmid for overexpression of protein with N-terminal His_6_-SUMO fusion, Amp^R^ | Bendezu et al., 2009 |
| pVGFPC-4 | Integrative vector for the production of fusion proteins carrying a C-terminal eGFP tag under the control of van, Gent^R^ | Thanbichler et al., 2007 |
| pXCHYC-2 | Integrative vector for the production of fusion proteins carrying a C-terminal mCherry tag under the control of P*_xyl_*, Kan^R^ | Thanbichler et al., 2007 |
| pXmVENC-2 | Integration plasmid for the production of fusion proteins carrying a C-terminal mVenus tag under the control of P*_xyl_*, Kan^R^ | (a) Site-directed mutagenesis of *venus* by inverse PCR using pXVENC-2 as template and primers venus-mut-for/-rev  (b) amplification of *venus*(A207K) from the mutagenized vector by PCR using primers Pxyl-GA-for and venus-GA-r2  (c) insertion of the PCR product into *Nde*I/*Nhe*I-treated pXVENC-2 by Gibson Assembly |
| pXmVENN-1 | Integration plasmid for the production of fusion proteins carrying an N-terminal mVenus tag under the control of P*_xyl_*, Strep/Spec^R^ | (a) Amplification of *mVenus* from pXmVENC-2 by PCR with primers oLY167 and oLY168  (b) Digestions of pXVENN-1 and the PCR product with *Nde*I and *Bsr*GI and subsequent ligation |
| pXmNeonGreenC-4 | Integration plasmid for the production of fusion proteins carrying a C-terminal mNenoGreen tag under the control of P*_xyl_*, Gent^R^ | (a) Amplification of *mNeonGreen* from pmNeonGreen-N1 (Allele Biotechnology) by PCR with primers oLY299 and oLY300  (b) Digestion of pXGFPC-4 and the PCR product with *Age*I and *Nhe*I and subsequent ligation |
|  |  |  |
