# Supplementary material for: Membrane binding properties of the cytoskeletal protein bactofilin
Source: eLife. 2025 Sep 19;13:RP100749. doi: 10.7554/eLife.100749 (PMC12448750; doi:10.7554/eLife.100749)
Supplement: MDAR checklist [file elife-100749-mdarchecklist1.docx]

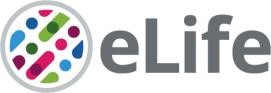


**Materials Design Analysis Reporting (MDAR)**

**Checklist for Authors**

The [MDAR framework](https://osf.io/xfpn4/) establishes a minimum set of requirements in transparent reporting mainly applicable to studies in the life sciences.

*eLife* asks authors to **provide detailed information within their article** to facilitate the interpretation and replication of their work. Authors can also upload supporting materials to comply with relevant reporting guidelines for health-related research (see [EQUATOR Network)](http://www.equator-network.org/), life science research (see the [BioSharing Information Resource)](http://biosharing.org/), or animal research (see the [ARRIVE Guidelines](http://www.plosbiology.org/article/info:doi/10.1371/journal.pbio.1000412) and the [STRANGE Framework;](https://doi.org/10.1038/d41586-020-01751-5) for details, see *eLife*’s [Journal Policies)](https://reviewer.elifesciences.org/author-guide/journal-policies). Where applicable, authors should refer to any relevant reporting standards materials in this form.

For all that apply, please note **where in the article** the information is provided. Please note that we also collect information about data availability and ethics in the submission form.

**Materials:**


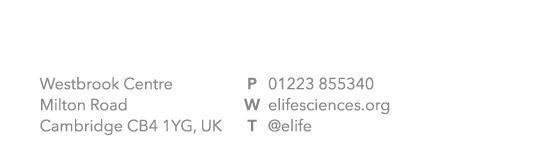


| **Newly created materials** | **Indicate where provided:**  **section/figure legend** | **N/A** |
| --- | --- | --- |
| The manuscript includes a dedicated "materials availability statement" providing transparent disclosure about availability of newly created materials including details on how materials can be accessed and describing any restrictions on access. | A material availability statement is included in the manuscript. |  |

| **Antibodies** | **Indicate where provided:**  **section/figure legend** | **N/A** |
| --- | --- | --- |
| For commercial reagents, provide supplier name, catalogue number and [RRID,](https://scicrunch.org/resources) if available. | The relevant information is given in the Materials section. |  |

| **DNA and RNA sequences** | **Indicate where provided:**  **section/figure legend** | **N/A** |
| --- | --- | --- |
| Short novel DNA or RNA including primers, probes: Sequences should be included or deposited in a public repository. | The sequences of the oligonucleotides used are provided in Supplementary file 7. |  |

| **Cell materials** | **Indicate where provided:**  **section/figure legend** | **N/A** |
| --- | --- | --- |
| Cell lines: Provide species information, strain. Provide accession number in repository OR supplier name, catalog number, clone number, OR RRID. |  | N/A |
| Primary cultures: Provide species, strain, sex of origin, genetic modification status. |  | N/A |

| **Experimental animals** | **Indicate where provided:**  **section/figure legend** | **N/A** |
| --- | --- | --- |
| Laboratory animals or Model organisms: Provide species, strain, sex, age, genetic modification status. Provide accession number in repository OR supplier name, catalog number, clone number, OR RRID. |  | N/A |
| Animal observed in or captured from the field: Provide species, sex, and age where possible. |  | N/A |

| **Plants and microbes** | **Indicate where provided:**  **section/figure legend** | **N/A** |
| --- | --- | --- |
| Plants: provide species and strain, ecotype and cultivar where relevant, unique accession number if available, and source (including location for collected wild specimens). |  | N/A |
| Microbes: provide species and strain, unique accession number if available, and source. | Information about the bacterial strains used in this study is provided in Supplementary file 5. | N/A |

| **Human research participants** | **Indicate where provided: section/figure legend) or state if these demographics were not collected** | **N/A** |
| --- | --- | --- |
| If collected and within the bounds of privacy constraints report on age, sex, gender and ethnicity for all study participants. |  | N/A |

**Design:**

| **Study protocol** | **Indicate where provided:**  **section/figure legend** | **N/A** |
| --- | --- | --- |
| If the study protocol has been pre-registered, provide DOI. For clinical trials, provide the trial registration number OR cite DOI. |  | N/A |

| **Laboratory protocol** | **Indicate where provided:**  **section/figure legend** | **N/A** |
| --- | --- | --- |
| Provide DOI OR other citation details if detailed step-by-step protocols are available. |  | N/A |

| **Experimental study design (statistics details) *** |  |  |
| --- | --- | --- |
| **For in vivo studies: State whether and how the following have been done** | **Indicate where provided:**  **section/figure legend. If it could have been done, but was not, write “not done”** | **N/A** |
| Sample size determination | For live-cell imaging, 100 or more cells were analyzed per strain. Numbers in this range are standard in the field and, in our hands, sufficient to ensure precise measurements. |  |
| Randomisation | See the “Statistics and reproducibility” section. |  |
| Blinding | Researchers were not blinded to the identity of the strains or proteins analyzed, as this was not technically or practically feasible. However, care was taken not to introduce any bias during data acquisition, and the same analysis pipeline was used across strains and proteins for each assay. |  |
| Inclusion/exclusion criteria | See the “Statistics and reproducibility” section. |  |

| **Sample definition and in-laboratory replication** | **Indicate where provided:**  **section/figure legend** | **N/A** |
| --- | --- | --- |
| State number of times the experiment was replicated in the laboratory. | When applicable, the number of replicates is indicated in the figure legends. |  |
| Define whether data describe technical or biological replicates. | Data describe biological replicates. |  |

| **Ethics** | **Indicate where provided: section/submission form** | **N/A** |
| --- | --- | --- |
| Studies involving human participants: State details of authority granting ethics approval (IRB or equivalent committee(s), provide reference number for approval. |  | N/A |
| Studies involving experimental animals: State details of authority granting ethics approval (IRB or equivalent committee(s), provide reference number for approval. |  | N/A |
| Studies involving specimen and field samples: State if relevant permits obtained, provide details of authority approving study; if none were required, explain why. |  | N/A |

| **Dual Use Research of Concern (DURC)** | **Indicate where provided: section/submission form** | **N/A** |
| --- | --- | --- |
| If study is subject to dual use research of concern regulations, state the authority granting approval and reference number for the regulatory approval. |  | N/A |

**Analysis:**

| **Attrition** | **Indicate where provided:**  **section/figure legend** | **N/A** |
| --- | --- | --- |
| Describe whether exclusion criteria were pre-established. Report if sample or data points were omitted from analysis. If yes, report if this was due to attrition or intentional exclusion and provide justification. | All data generated or analyzed are included in the figures. |  |

| **Statistics** | **Indicate where provided:**  **section/figure legend** | **N/A** |
| --- | --- | --- |
| Describe statistical tests used and justify choice of tests. | See the “Statistics and reproducibility” section in the Methods. |  |

| **Data availability** | **Indicate where provided: section/submission form** | **N/A** |
| --- | --- | --- |
| For newly created and reused datasets, the manuscript includes a data availability statement that provides details for access (or notes restrictions on access). | All data associated with this study are present in the paper or the Supplementary material. |  |
| When newly created datasets are publicly available, provide accession number in repository OR DOI and licensing details where available. |  | N/A |
| If reused data is publicly available provide accession number in repository OR DOI, OR URL, OR citation. |  | N/A |

| **Code availability** | **Indicate where provided:**  **section/figure legend** | **N/A** |
| --- | --- | --- |
| For any computer code/software/mathematical algorithms essential for replicating the main findings of the study, whether newly generated or re-used, the manuscript includes a data availability statement that provides details for access or notes restrictions. |  | N/A |
| Where newly generated code is publicly available, provide accession number in repository, OR DOI OR URL and licensing details where available. State any restrictions on code availability or accessibility. |  | N/A |
| If reused code is publicly available provide accession number in repository OR DOI OR URL, OR citation. | The relevant information is provided in the Materials section. |  |

**Reporting:**

The MDAR framework recommends adoption of discipline-specific guidelines, established and endorsed through community initiatives.

| **Adherence to community standards** | **Indicate where provided:**  **section/figure legend** | **N/A** |
| --- | --- | --- |
| State if relevant guidelines (e.g., ICMJE, MIBBI, ARRIVE, STRANGE) have been followed, and whether a checklist (e.g.,  CONSORT, PRISMA, ARRIVE) is provided with the manuscript. |  | N/A |
